# Development of a genome-wide InDel marker set for allele discrimination between rice (*Oryza sativa*) and the other seven AA-genome *Oryza* species

**DOI:** 10.1038/s41598-021-88533-9

**Published:** 2021-04-26

**Authors:** Sherry Lou Hechanova, Kamal Bhattarai, Eliza Vie Simon, Graciana Clave, Pathmasiri Karunarathne, Eok-Keun Ahn, Charng-Pei Li, Jeom-Sig Lee, Ajay Kohli, N. Ruaraidh Sackville Hamilton, Jose E. Hernandez, Glenn B. Gregorio, Kshirod K. Jena, Gynheung An, Sung-Ryul Kim

**Affiliations:** 1 Gene Identification and Validation Group, Genetic Design and Validation Unit, International Rice Research Institute (IRRI), 4031 Los Baños, Laguna Philippines; 2 Institute of Crop Science (ICropS), College of Agriculture and Food Science, University of the Philippines Los Baños (UPLB), 4031 Los Baños, Laguna Philippines; 3 National Institute of Crop Science, Rural Development Administration (RDA), Suwon, 16429 Republic of Korea; 4 Taiwan Agricultural Research Institute (TARI), Council of Agriculture, Taichung City, Taiwan; 5 Crop Biotech Institute and Graduate School of Biotechnology, Kyung Hee University, Yongin, Republic of Korea; 6 School of Biotechnology, KIIT Deemed University, Bhubaneswar, Odisha India

**Keywords:** Plant sciences, Plant breeding, Plant genetics

## Abstract

Wild relatives of rice in the genus *Oryza* (composed of 24 species with 11 different genome types) have been significantly contributing to the varietal improvement of rice (*Oryza sativa*). More than 4000 accessions of wild rice species are available and they are regarded as a “genetic reservoir” for further rice improvement. DNA markers are essential tools in genetic analysis and breeding. To date, genome-wide marker sets for wild rice species have not been well established and this is one of the major difficulties for the efficient use of wild germplasm. Here, we developed 541 genome-wide InDel markers for the discrimination of alleles between the cultivated species *O. sativa* and the other seven AA-genome species by positional multiple sequence alignments among five AA-genome species with four rice varieties. The newly developed markers were tested by PCR-agarose gel analysis of 24 accessions from eight AA genome species (three accessions per species) along with two representative cultivars (*O. sativa* subsp. *indica* cv. IR24 and subsp. *japonica* cv. Nipponbare). Marker polymorphism was validated for 475 markers. The number of polymorphic markers between IR24 and each species (three accessions) ranged from 338 (versus *O. rufipogon*) to 416 (versus *O. longistaminata*) and the values in comparison with Nipponbare ranged from 179 (versus *O. glaberrima*) to 323 (versus *O. glumaepatula*). These marker sets will be useful for genetic studies and use of the AA-genome wild rice species.

## Introduction

Crop improvement by breeding relies on genetic variation. Domestication processes from wild progenitors and repeated use of elite germplasm in breeding programs narrowed down the genetic variation^1,2^^.^ In addition, the favorable alleles of some major identified genes governing important agronomic traits are already present in many modern rice varieties^3^, indicating that these alleles are not effective in breeding programs. Exploring diverse germplasm and identifying rare/low-frequency alleles are required for further crop improvement. Wild relatives of rice have been regarded as genetic reservoirs for rice improvement. They have been surviving in diverse environments worldwide such as riversides, swamps, forests, and seashores without any protection for millions of years, suggesting that their genomes evolved to cope with their given environments and environmental changes. Many useful traits were mined from wild rice relatives, including abiotic stress tolerance (salinity, heat, drought, iron toxicity, P-deficiency, aluminum toxicity), biotic stress resistance (bacterial leaf blight, sheath blight, blast, rice yellow mottle virus, brown planthopper, white-backed planthopper, grassy stunt virus), and yield-related traits^4–7^ as well as novel traits, which are absent in the cultivated species, such as long-exserted stigma for increasing the outcrossing rate in hybrid seed production^8^ and early-morning flowering for avoiding heat stress during pollination-fertilization processes^9^. Furthermore, an interspecific hybridization between the cultivated species and wild species was conducted for the selection of improved lines for breeding purposes and the identification of the QTLs/genes governing important agronomic traits by applying genetics and molecular genomics tools. As consequences of those efforts, the DNA of wild species knowingly and unknowingly contributed a lot to rice improvement such as for biotic stress resistance, abiotic stress tolerance, and grain yield. Especially, many biotic stress resistance QTLs/genes for bacterial leaf blight, blast, and brown planthopper (BPH) were identified from the wild species and they were widely used in rice breeding programs for local elite rice varietal improvement^10–13^. However, vast allelic variation and novel genetic factors from wild species remain untapped. The novel genes/alleles from exotic germplasm and wild rice relatives might be rare alleles that are not present in cultivated rice and they could be effective in most rice, including modern varieties.

Wild rice species and the cultivated rice species belong to the genus *Oryza*, which consists of two cultivated species, *O. sativa* (Asian rice) and *O. glaberrima* (African rice), and 22 wild species representing 11 genome types, of which six are diploid (AA, BB, CC, EE, FF, and GG) and five are allotetraploid (BBCC, CCDD, HHJJ, HHKK, and KKLL)^14,15^. Based on the degree of crossability with the cultivated species, these wild species are classified into primary, secondary, and tertiary genepools. Among these genepools, the primary genepool contains the two cultivated species and six wild species (*O. barthii*, *O. longistaminata*, *O. nivara*, *O. glumaepatula*, *O. meridionalis,* and *O. rufipogon*) that shared the AA genome and thus can more easily produce interspecific hybrids and their progenies between the cultivated species and the other six AA-genome-type wild species than the other genepools. *O. glaberrima* was domesticated from *O. barthii* and it has been cultivated mainly on the African continent. The major cultivated species *O. sativa*, which has been cultivated in Asian countries and is extended globally, consists of two subspecies, *indica* and *japonica*, which were domesticated from their wild progenitors *O. nivara* and *O. rufipogon*, respectively^16^.

DNA markers play important roles in many genetic research and breeding programs, including assessment of genetic diversity, identification of QTLs, gene mapping, marker-assisted tagging of target QTLs/genes, and characterization of alien introgression lines from wild species of rice. To discriminate DNA variation (nucleotide substitution or insertion/deletion), several kinds of DNA markers were developed over the past decades, such as restriction fragment length polymorphism (RFLP) by using restriction enzymes and DNA hybridization^17^, rapid amplified polymorphic DNA (RAPD)^18^, sequence-tagged site (STS)^19^, cleaved amplified polymorphic sequence (CAPS)^20^, tetra-primer and dominant PCR markers^21^, and microsatellites/SSRs (simple sequence repeats)^22–25^. In addition, next-generation sequencing (NGS) and advanced genotyping technologies enabled high-throughput single nucleotide polymorphism (SNP) genotyping such as Fluidigm Dynamic Arrays, Douglas Scientific Array Tape, LGC KASP markers, Illumina Infinium SNP genotyping platform, and genotyping by sequencing (GBS)^26–28^. Although SNP genotyping technology is available, PCR and gel-based InDel markers have practical value for researchers and breeders because of their technical simplicity and ease of accessibility. Hence, to date, InDel markers are still widely used in genetic analysis and breeding. Currently, a few sets of genome-wide InDel markers discriminating the two subspecies alleles (*indica* and *japonica*) in *O. sativa* have been developed and are publically available^29–32^ but these markers are not suitable for discriminating cultivated and wild species alleles. Some InDel markers that can discriminate the alleles between the cultivated species and closely related wild species have been reported^33,34^ but the limited numbers of markers showed polymorphism (43–91 markers) except for the comparisons between the cultivars and *O. rufipogon* (96–155 markers) throughout the 12 rice chromosomes. Orjuela et al.^35^ developed 165 anchors consisted of 489 SSR markers which had high potential of polymorphisms among the AA-genome species and they validated the markers with high frequency of polymorphism (86.2%). But the PCR products were analyzed by PAGE with silver staining which is a laborious method with low throughput. Thus, the SSR markers with agarose-gel analysis are still highly used to detect wild rice introgressions in *O. sativa* backgrounds^36–39^ although screening of large numbers of SSR markers in experimental materials is required and limited polymorphic markers are obtained.

Hence, the objectives of this study are to develop a genome-wide InDel marker set that discriminates the alleles between the major cultivated species (*O. sativa*) and the other seven AA-genome species in agarose gel and to validate the newly developed markers using two representative cultivars (Nipponbare and IR24) together with 24 accessions from eight AA-genome species (three accessions per species) and the early generation lines of interspecific hybrids. A medium-density InDel marker set for AA-genome *Oryza* species was successfully developed and the markers were validated. The information produced in this study will provide groups of marker sets consisting of the potential polymorphic markers between *indica* and specific AA-genome species as well as between *japonica* and specific AA-genome species.

## Materials and methods

### Sequence preparation from public databases

The chromosome levels of whole-genome sequences of AA-genome wild species and *indica* cultivars were obtained from the National Center for Biotechnology Information (NCBI, https://www.ncbi.nlm.nih.gov/) with GenBank accession numbers: *O. nivara* (PRJNA48107), *O. barthii* (PRJNA30379), *O. glumaepatula* (PRJNA48429), and *O. meridionalis* (PRJNA48433), which were submitted by the International *Oryza* Map Alignment Project (IOMAP)^40^; Minghui 63 (PRJNA302543) and Zhenshan 97 (PRJNA302542) submitted by Zhang et al.^41^; and IR8 (PRJNA353946) submitted by Stein et al.^16^. The whole-genome sequence of *O. longistaminata* was obtained from the link http://www.olinfres.nig.ac.jp/^42^.

### Sequence comparisons among species

Bait sequences (30‒100 kb) were obtained from the rice reference genome sequence (*O. sativa* subsp. *japonica* cv. Nipponbare) at RAP-DB (https://rapdb.dna.affrc.go.jp/). We avoided repeat regions such as transposable elements in bait sequence preparation and manually selected locations from top to end at approximately 1-Mb intervals on each chromosome. For isolation of the orthologous regions to the bait sequences, we used the Genomic Aligner (NG Aligner) tool embedded in NCBI Genome Workbench software (https://www.ncbi.nlm.nih.gov/tools/gbench/). Each chromosome sequence from five wild rice species and three *indica* varieties obtained from the public databases described above was loaded into the software together with the bait sequence. The orthologous region in each species/variety for the bait sequence was manually obtained one by one using NG Aligner. Multiple sequence alignments were conducted with the extracted orthologous sequences together with the bait sequence using the web-based tool mVISTA (http://genome.lbl.gov/vista/)^43,44^. The aligned sequences were loaded into BioEdit software^45^ for better visualization of InDel regions.

### Primer design

InDel regions were manually screened at the multiple sequence alignments in 30‒100-kb ranges. One or two InDel regions showing a > 20-bp gap between the cultivars and all wild species or between some cultivars and some wild species were selected. After selection of the InDel for marker development, forward and reverse primers were manually designed for normal PCRs (annealing temperature 55 °C and PCR product sizes 100‒500 bp). Redundancy of the primer sequences was checked by BLAST in the RAP-DB and a unique hit was selected for the primer sequence.

### Plant materials and growth

For validation of the newly developed markers, we selected varieties IR24 and Nipponbare as the representative background parents of *O. sativa* subsp. *indica* and subsp. *japonica* type rice, respectively. Regarding the selection of germplasm from six wild AA-genome species and *O. glaberrima* (African rice), we selected three accessions per species based on the geographical long distance within a species (Table 1). In addition, we included three temperate *japonica* varieties selected in the same way for checking the polymorphism between *indica* variety IR24 and the *japonica* varieties. Seeds were obtained from the International Rice Genebank (https://www.irri.org/international-rice-genebank) at the International Rice Research Institute (IRRI) and they were grown in a glasshouse for wild species at IRRI headquarters, Los Baños, Philippines. For further validation of the markers, which showed monomorphism among the above 26 accessions of AA genome species (IR24, Nipponbare, 18 from wild rice species, three from *O. glaberrima*, and three from *japonica* varieties), the markers were applied for the additional 11 popular varieties: two *japonica* varieties (Ilpumbyeo and Tainung 71) and nine *indica* varieties (IR8, IR64, IRRI123, IRRI154, Milyang 23, Zhenshan 97B, Kasalath, Minghui 63, and Samba Mahsuri).

**Table 1:** List of AA-genome germplasm used in this study for experimental marker validation.

Code	Species/variety	Accession no. (IRRI Genebank)	Origin
IR24	*O. sativa* subsp. *indica* var. IR24	–	Philippines
NB	*O. sativa* subsp. *japonica* var. Nipponbare	–	Japan
Bart_A01	*O. barthii*	IRGC 105613	Botswana
Bart_A02	*O. barthii*	IRGC 104124	Chad
Bart_A03	*O. barthii*	IRGC 106291	Mauritania
Glab_A04	*O. glaberrima*	IRGC 102486	Liberia
Glab_A05	*O. glaberrima*	IRGC 101879	Nigeria
Glab_A06	*O. glaberrima*	IRGC 103458	Senegal
Glum_A07	*O. glumaepatula*	IRGC 105661	Brazil
Glum_A08	*O. glumaepatula*	IRGC 105561	Colombia
Glum_A09	*O. glumaepatula*	IRGC 100184	Cuba
Long_A10	*O. longistaminata*	IRGC 81967	Botswana
Long_A11	*O. longistaminata*	IRGC 105200	Ethiopia
Long_A12	*O. longistaminata*	IRGC 101754	Senegal
Meri_A13	*O. meridionalis*	IRGC 105290	Australia
Meri_A14	*O. meridionalis*	IRGC 103319	Australia
Meri_A15	*O. meridionalis*	IRGC 105300	Australia
Niva_A16	*O. nivara*	IRGC 106495	India
Niva_A17	*O. nivara*	IRGC 103837	Bangladesh
Niva_A18	*O. nivara*	IRGC 93196	Nepal
Rufi_A19	*O. rufipogon*	IRGC 106276	Papua New Guinea
Rufi_A20	*O. rufipogon*	IRGC 105491	Malaysia
Rufi_A21	*O. rufipogon*	IRGC 93210	Nepal
Japo_A22	*O. sativa* subsp. *japonica* var. 7516-14	IRGC 132271	Japan
Japo_A23	*O. sativa* subsp. *japonica* var. Rocca	IRGC 125888	Italy
Japo_A24	*O. sativa* subsp. *japonica* var. WIR 1878	IRGC 121543	Kazakhstan

### Generation of F_1_, BC_1_F_1_, and BC_2_F_1_ plants

We used *indica* rice variety IR24 as a recurrent parent for marker validation in the interspecific hybrid plants destined for further development of introgression lines (ILs) or chromosome segment substitution lines (CSSLs) in the following generations. IR24 bred by IRRI was frequently used as an elite parental line for many *indica* rice breeding programs^46^ as well as being used as the recurrent line for the development of near-isogenic lines for bacterial leaf blight resistance^47^ and brown planthopper resistance genes^48^. We made crosses between IR24 (female) and other AA-genome species (male donors) in the glasshouse at IRRI. F_1_ plants were obtained through embryo rescue described by Jena et al.^49^. After the selection of true interspecific F_1_ plants by marker applications, BC_1_F_1_ and BC_2_F_1_ plants were generated through backcrossing.

### DNA preparation and PCR analysis

Genomic DNA was prepared by using the simple DNA preparation method described by Kim et al.^50^, which does not require phenol/chloroform extraction and isopropanol precipitation steps. Briefly, a small piece (2‒4 cm long) of fresh leaf from each plant material was directly collected in a 2-mL tube containing two steel balls. After freezing the tubes in liquid nitrogen, the samples were ground using a 2010 Geno/Grinder (http://www.spexsampleprep.com). In each tube, 200 µL of TPE buffer (100 mM Tris–HCl pH 9.5, 1 M KCl, 10 mM EDTA pH 8.0) were added and the samples were incubated at 65 °C for 30 min. After incubation, the samples were diluted by adding 1 mL of double-distilled water and centrifuged for 15 min at the maximum speed. The supernatant (genomic DNA) was transferred to a new 96-well plate and stored at 4 °C for PCR analysis. The 20-µL PCR mixture contained 1 × PCR buffer, 200 μM of each dNTP, 0.25 μM of each primer, 2 µL of leaf extract prepared by the above TPE method, and 1 unit of BioFact *Taq* DNA polymerase (http://bio-ft.com/en). Thermal cycles were programmed as follows: 94 °C for 3 min; 35 cycles of 95 °C for 25 s, 55 °C for 25 s, and 72 °C for 60 s; and 72 °C for 5 min. The PCR products were separated in 2.5‒4.0% agarose gel with 0.5× TBE buffer. For clear comparisons of PCR product sizes between the cultivated rice and AA-genome species as well as between parents and their progenies, the PCR products were loaded at the same lanes in an agarose gel. Gel images were cropped for each marker and presented in the manuscript.

### Graphical mapping of the markers on the rice genome

The physical locations of the markers were mapped on the 12 rice chromosomes by using the web-based tool PhenoGram (http://visualization.ritchielab.psu.edu/)^51^ and then each chromosome was manually rearranged from top to bottom based on a ruler.

### Ethical approval statement

All experiments conducted and reported in this manuscript were carried out following relevant guidelines and regulations of the government of the Philippines and of the International Rice Research Institute.

## Results

### Establishment of a strategy for the development of a genome-wide InDel marker set

**Figure 1:**
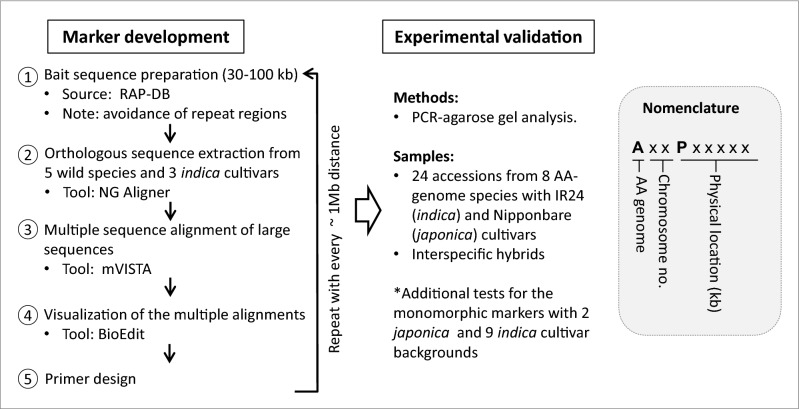
Strategy for the development of a genome-wide InDel marker set for allele discrimination between *O. sativa* and other AA-genome species in the genus *Oryza*.

In order to develop high-quality InDel markers that can discriminate the alleles between the major cultivated rice (Asian rice) and the other AA-genome species in the *Oryza* genus, we intended (i) evenly distributed markers throughout the 12 chromosomes (~ 1-Mb interval between neighboring markers), (ii) avoidance of duplicated/repeat regions in the rice genome, (iii) clear separation of the alleles in agarose gel, (iv) even PCR amplification efficiency among germplasm/varieties through selection of the conserved sequences for primer annealing sites, and (v) user-friendly nomenclature and data summarization. For this, we established a marker development strategy as follows: (1) preparation of bait sequences from RAP-DB, (2) orthologous sequence extraction from the genome sequences of five wild species and three *indica* cultivars, (3) multiple sequence alignments among the orthologous sequences, (4) visualization of the multiple alignments, (5) primer design, and (6) experimental validation by PCR-agarose gel analysis (Fig. 1). In bait sequence preparation, we started at the tip of each chromosome by using the genome browser at RAP-DB and we selected 30‒100-kb length as a bait sequence. This long bait sequence eventually will be aligned together with the orthologous sequences, resulting in providing more choices for selection of the best InDel for marker design in terms of numbers of polymorphism among the aligned sequences and gap sizes (> 20 bp). We also considered avoiding long repeat sequences such as transposon/retrotransposon sequences in bait sequence selection so that the markers could be targeted at a unique locus in the genome. The orthologous sequences for the bait sequence were extracted by the NG Aligner tool from five wild species and three *indica* cultivars. We could not obtain the corresponding sequences in some samples. This might be due to the absence of the corresponding region or poor sequence assembly quality. When we obtained more than five sequences from the eight sequences, we performed multiple sequence alignments by using the web-based mVISTA tool. The aligned sequences were imported into BioEdit software for highlighting sequence polymorphism such as InDel and SNP. Then, the good InDels showing polymorphism between the cultivars and all wild species or between some cultivars (mostly common InDels among the three *indica* reference sequences from Minghui 63, Zhenshan 97, and IR8) and some wild species with a > 20-bp gap were selected for marker designing. The conserved regions surrounding the selected InDels among the aligned sequences were used for primer design so that the primers could be annealed properly to all the species/varieties for even PCR amplification efficiency among the alleles. The same procedures were repeated at ~ 1-Mb intervals in each chromosome except for the long repeat regions such as centromeres. We successfully designed 541 InDel markers in total throughout the 12 rice chromosomes based on this strategy. Each marker was referred to as “AxxPxxxxx” (x = digit) (Fig. 1): “A” stands for genome type A, the following 2-digit code means chromosome number, and the “P with 5 digits” indicates the physical location of the marker (kb) at the reference genome sequence IRGSP1.0. For example, A01P00302 and A12P27058 are located at the 0.302-Mb locus on chromosome 1 and at the 27.058-Mb locus on chromosome 12, respectively. This naming system allows for the clear identification of the physical location of the markers.

### Experimental validation of the newly developed InDel markers

The 541 newly developed InDel markers were tested with two representative cultivars of *O. sativa* (subsp. *indica* cv. IR24 and subsp. *japonica* cv. Nipponbare) and 24 accessions from eight AA-genome species (three accessions per species; Table 1) by PCR-agarose gel analysis. Out of the 541 markers, 466 markers successfully exhibited polymorphism between the rice cultivars (IR24 or Nipponbare) and the other AA-genome species. Among the remaining markers, 16 showed a monomorphic band among 26 accessions and 59 markers exhibited either no/weak amplification or unexpected extra bands. For the monomorphic markers, there is still a possibility of polymorphism in other cultivar backgrounds. Thus, the 16 monomorphic markers were tested by using 11 popular rice varieties consisting of 2 *japonica* varieties (Ilpumbyeo and Tainung 71) and 9 *indica* varieties (IR8, IR64, IRRI123, IRRI154, Milyang 23, Zhenshan 97B, Kasalath, Minghui 63, and Samba Mahsuri). We found that 9 out of 16 showed polymorphism (Supplementary Fig. S1). In total, 475 markers (87.8%) were experimentally validated. The information on the marker set is summarized in Supplementary Table S1.

**Figure 2:**
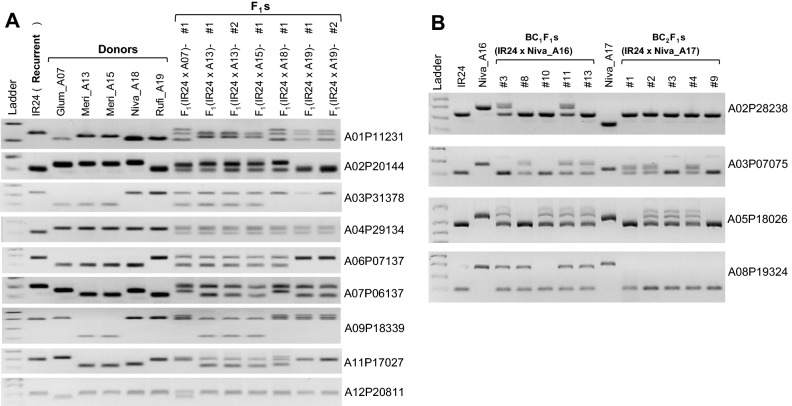
Applications of the newly developed markers in the early generations of hybrids between IR24 and wild species. (**A**) Test of the markers in F_1_ plants together with both recurrent (IR24) and donor parents. (**B**) Test of the markers in BC_1_F_1_ and BC_2_F_1_ plants derived from accessions Niva_A16 and Niva_A17.

In addition to parental lines, we applied the nine selected polymorphic markers to seven interspecific hybrids between IR24 and wild rice species such as *O. glumaepatula* (Glum_A07), *O. meridionalis* (Meri_A13 and A15), *O. rufipogon* (Rufi_A19), and *O. nivara* (Niva_A16, A17, and A18). All the F_1_ plants followed the genotype of their parents (IR24 and/or the wild species) for all the markers tested, as expected. In cases of the polymorphic markers between parents, the markers clearly exhibited both alleles (PCR bands) in the F_1_s (Fig. 2A). The assays with new markers showed that all seven F_1_ plants are true hybrids, demonstrating that the markers are useful for checking F_1_ hybridity. Wild rice introgression between IR24 and two accessions of *O. nivara* (Niva_A16 and A17) at BC_1_F_1_ and BC_2_F_1_ generations was studied with four markers located at different chromosomes (Fig. 2B). Three markers, except for A05P18026, also showed polymorphism between accessions Niva_16 and Niva_17 (multi-allele markers), which will be useful for discriminating accessions within the same species while we are handling many accessions. The markers tested showed clear band separation between/among three parental lines and the test results in the progenies revealed which chromosomes and loci were introgressed in each BC_1_F_1_ and BC_2_F_1_ plant (for example, BC_1_F_1_(IR24/Niva_A16)-#11 plant possessed four introgressions at least, whereas plant #8 had only one introgression at the 7.075-Mb region of chromosome 3 (A03P07075)) (Fig. 2B). This result supports the newly developed marker sets being useful for discriminating species and accessions as well as for detecting wild introgressions.

### Number of polymorphic markers and their distribution across the genome

**Figure 3:**
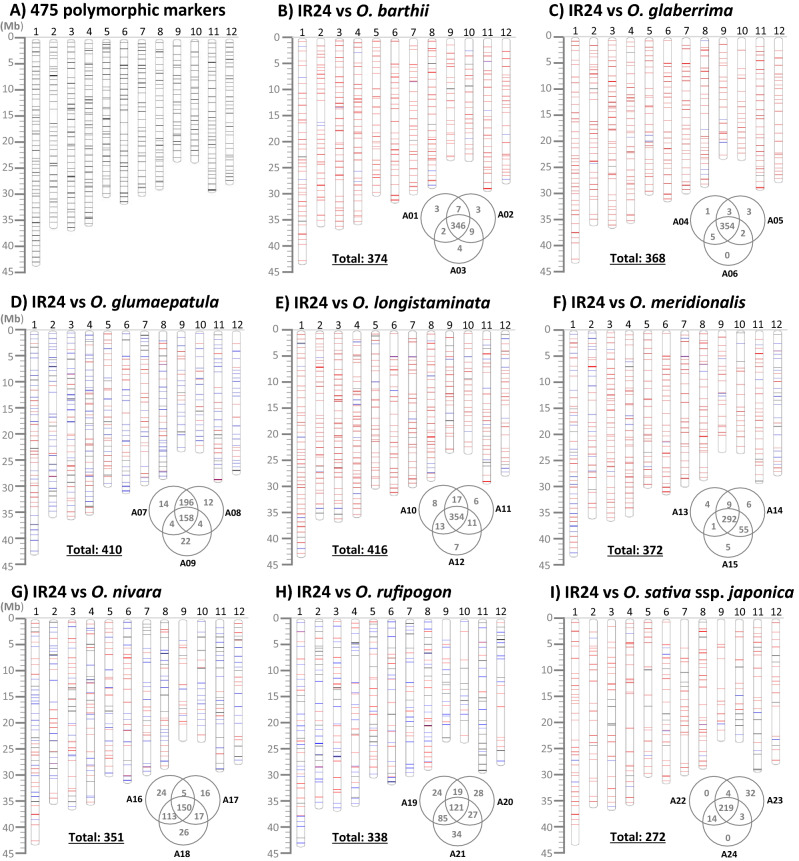
Physical locations of the polymorphic markers. The position of each marker was mapped on the rice reference genome (Os-Nipponbare-Reference-IRGSP-1.0) with a horizontal bar. (**A**) All available 475 polymorphic markers between *O. sativa* and the other AA-genome species. The selected polymorphic markers showing polymorphism between IR24 and *O. barthii* (**B**), *O. glaberrima* (**C**), *O. glumaepatula* (**D**), *O. longistaminata* (**E**), *O. meridionalis* (**F**), *O. nivara* (**G**), *O. rufipogon* (**H**), and *O. sativa* subsp. *japonica* (**I**), respectively. Within a species, the common polymorphic markers among three accessions and between two accessions are highlighted by red and blue bars, respectively, and the accession-specific polymorphic markers are depicted with a black bar.

To observe the distribution degree of the 475 validated markers, we mapped the physical location of each marker on the rice genome. They were evenly distributed across the 12 chromosomes with an average of ~ 787-kb intervals between neighboring markers. Except for four loci on chromosomes 2, 6, and 10, the marker intervals were less than 2 Mb in size (Fig. 3A). We estimated the number of polymorphic markers between IR24 and each accession from the other seven species based on the genotype scoring data of the 475 markers. The average number of polymorphic markers between IR24 and 21 accessions is 323.0. The marker number ranges from 187 (IR24 vs Glum_A09) to 391 (IR24 vs Long_A11) (Table 2). Based on these results, we made groups of polymorphic marker sets at the species level for easy selection of potential polymorphic markers based on the species. Briefly, all the polymorphic markers were collected from three accessions in each species and then the accession-specific polymorphic markers and the common polymorphic markers between two accessions or among three accessions were identified and listed (Supplementary Tables, Table S2: *O. barthii*, Table S3: *O. glaberrima*, Table S4: *O. glumaepatula*, Table S5: *O. longistaminata*, Table S6: *O. meridionalis*, Table S7: *O. nivara*, and Table S8: *O. rufipogon*). The available polymorphic markers for each species were calculated (shown by a Venn diagram) and mapped on the rice genome (Fig. 3B–H). The number of markers ranges from 338 (IR24 vs *O. rufipogon*) to 416 (IR24 vs *O. longistaminata*). These markers will be useful for genetic analysis and breeding purposes between *indica* cultivars and other AA-genome species. We also surveyed the polymorphic markers between IR24 and three accessions of *japonica* varieties (Supplementary Table S9) and we obtained 272 markers without redundancy (Fig. 3I).

**Table 2:** Number of polymorhpic markers and the frequency of heterozygosity.

Samples	No. of polymorphic markers		No. of 2-band markers (%)	
	IR24 vs	NB vs		
Bart_A01	358	174	1	0.211
Bart_A02	365	174	1	0.211
Bart_A03	361	175	1	0.211
Glab_A04	363	174	3	0.632
Glab_A05	362	176	2	0.421
Glab_A06	361	172	2	0.421
Glum_A07	374	179	1	0.211
Glum_A08	369	178	3	0.632
Glum_A09	187	212	18	3.789
Long_A10	390	239	60	12.632
Long_A11	391	243	60	12.632
Long_A12	384	236	44	9.263
Meri_A13	306	168	4	0.842
Meri_A14	359	203	2	0.421
Meri_A15	356	204	6	1.263
Niva_A16	291	126	1	0.211
Niva_A17	189	191	4	0.842
Niva_A18	306	138	4	0.842
Rufi_A19	249	163	18	3.789
Rufi_A20	189	179	3	0.632
Rufi_A21	273	149	36	7.579
Japo_A22	237	43	2	0.421
Japo_A23	256	39	2	0.421
Japo_A24	238	43	2	0.421

By the same analysis, the polymorphic markers between a *japonica* rice variety (Nipponbare) and 21 accessions of AA-genome species were analyzed. The average number of polymorphic markers between Nipponbare and 21 accessions is 187.4 and it ranges from 126 (Nipponbare vs Niva_A16) to 243 (Nipponbare vs Long_A11) (Table 2). The polymorphic markers between Nipponbare and the other seven species were selected respectively and listed in Supplementary Tables S10 to S16. Distributions of the markers were presented on the rice genome maps (Supplementary Fig. S2A–G). A survey of the polymorphic markers between Nipponbare and three *japonica* varieties revealed that only 64 markers out of 475 showed polymorphism (Supplementary Table S17 and Supplementary Fig. S2H). This result indicates that a high genome similarity exists among the *japonica* varieties although the three accessions originated from far distances (Table 1).

### Heterozygosity among the accessions tested

During marker validation of 24 accessions of AA genome species, we observed that some markers produced two clearly different sizes of PCR bands in some accessions (Supplementary Fig. S3), although all the accessions are not cross-derived materials. The double bands might be caused by heterozygous alleles at the specific locus or duplication of the marker sequences in the genome. The frequency of markers generating two bands was very low (one to four cases: 0.21‒0.84%) in most of the accessions tested (Table 2). However, all three accessions of *O. longistaminata* showed the highest frequency (44‒60 markers: 9.26‒12.63%). Two accessions of *O. rufipogon* (Rufi_A19 and A21) and one accession of *O. glumaepatula* (Glum_A09) also exhibited a high frequency of double bands (18‒36 markers: 3.79‒7.58%).

## Discussion

Yield increases and stable high yield of rice are crucial for global food security along with the world population increase. Climate changes as well as the prevalence of climate change-induced novel pathogens also threaten stable rice production. Thus, scientists and breeders have been trying to find some solutions from wild species of crops. Wild rice species already proved their genetic ability for rice improvement and are believed to be a “genetic reservoir” for further improvement of elite rice varieties. Although more than 4000 accessions of wild rice species have been collected and are maintained in the International Rice Genebank (https://www.irri.org/international-rice-genebank), only small portions of them have been used for breeding programs. These are highly valuable for obtaining novel genes, superior alleles, rare alleles, and the genes that are absent in the cultivated species. Although wild rice species themselves are valuable, they are far from having their genetic factors used for rice improvement in terms of breeding aspects. To use the genetic factors governing valuable traits, somehow the genetic factors (DNA) of wild species need to be transferred to the cultivated species through hybridization between *O. sativa* and wild species and following crossover-based DNA introgressions. The wild introgression lines in cultivar backgrounds such as CSSLs are regarded as “ready-to-use genetic materials” for varietal improvement^4,52^ because the background genome of the ILs is already close to the cultivars and it is efficient to identify the genetic factors associated with the acquired traits, which are absent/inferior in the recurrent cultivar backgrounds. Hence, the development of ILs and CSSLs is a good strategy for novel gene identification and its prompt transfer to elite varieties in relation to a rapid response to climate changes and consumer demand such as for nutrient-rich rice. For these reasons, many efforts have been made to develop ILs and CSSLs by using wild species^36–39,53^. However, to date, there are no suitable genome-wide marker sets that can discriminate the alleles between the cultivated species and wild species. Hechanova et al.^54^ developed 94 markers for CC-genome species by using the bacterial artificial chromosome (BAC) end sequences of *O. officinalis* having the CC genome but polymorphism for AA-genome species was not tested. Yamaki et al.^34^ developed 22 InDel markers to discriminate all genome types in the genus *Oryza* by using the BAC end sequences of 12 *Oryza* species but these were insufficient in breeding and genetics. In order to select polymorphic markers between *japonica* and AA-genome wild species, Niihama et al.^33^ applied 188 *indica*/*japonica* polymorphic InDel markers to 14 accessions from five AA-genome species and they were able to select high numbers of polymorphic markers (111‒153) only with *O. rufipogon* and not with the other four species (*O. barthii*, *O. glumaepatula*, *O. longistaminata*, and *O. meridionalis*) (only 68‒91 markers across the rice genome). In addition, many markers did not show PCR amplicons except for *O. rufipogon*, especially in the relatively distant species *O. longistaminata* and *O. meridionalis* (60‒85 markers), suggesting that the marker primers were not properly annealed in the wild species because of sequence variations at the primer binding sites or complete absence of the region in distant species. So, the RM markers developed for genetic analysis and breeding of the cultivated species are still commonly used for wild rice species as well. These markers were massively developed by using the sequence information from cultivated rice to amplify SSRs, which showed high potential of polymorphism caused by variation in the number of SSRs between/among germplasm^23,24^. Hence, scientists and breeders should screen the RM markers for their own plant materials to select polymorphic markers. This process is costly, time-consuming, and laborious and sometimes it is difficult to obtain enough markers with proper distribution/location. In addition, some polymorphic markers with small gaps (< 20 bp) between/among alleles are not clearly resolved in agarose gel. Although they can be separable in polyacrylamide gel electrophoresis (PAGE) showing high-resolution band separations, the use of PAGE is decreasing because of its more cumbersome procedures such as gel preparation and PCR product loading with lower throughput than in agarose gel analysis. Most of all, a high portion of RM markers used to fail to amplify in distant AA-genome species such as non-progenitors of cultivated rice and other genome types in the genus *Oryza*. Thus, we intended to systematically develop a genome-wide marker set for discrimination between the major cultivated species *O. sativa* and the other AA-genome species. We used publically available genome sequences of five wild species and we did positional multiple sequence alignments among five wild rice species with four cultivars, and we manually selected large InDel regions that can be resolved in agarose gel for marker development. We also checked the internal sequences to avoid difficult PCR regions such as high-GC regions. The primer sequences were selected in the conserved region among the aligned sequences so that the primers could be properly annealed to all the AA-genome species. Based on these schemes, we designed 541 markers across the 12 rice chromosomes and we successfully validated 475 markers showing polymorphism through PCR-agarose gel analysis in 21 accessions of AA-genome species. Most of the markers successfully amplified the target InDels and showed many polymorphisms even in the relatively distant species such as *O. longistaminat*a and *O. meridionalis* (Table 2). In addition, the locations of the markers were targeted with ~ 1-Mb intervals, resulting in even distribution across the rice genome (Fig. 3). Both the number of polymorphic markers and their distribution across the rice genome are suitable for genetic analysis and breeding.

For discrimination between *indica* and *japonica* alleles within *O. sativa*, a few sets of genome-wide markers were developed and they are publically available. Shen et al.^30^ extracted 479,406 InDel regions through genome sequence comparison between Nipponbare (*japonica*) and 93-11 (*indica*) and Liu et al.^29^ identified 2,329,544 InDels in 1767 rice genomes. However, only 108 and 100 InDel markers were developed from the extracted InDel regions and experimentally validated, respectively. Wu et al.^32^ developed 506 InDel markers based on the published rice genome sequences and they validated polymorphism from only 133 markers between Taiken 2 (*japonica*) and Taichung Sen 10 (*indica*) by PCR-agarose gel analysis. Recently, Hu et al.^31^ extracted 19,937 large InDel markers (30‒55-bp gap) based on two high-quality *indica* rice and one *japonica* rice reference genome sequences and they experimentally validated 346 markers in a panel of 22 cultivars by running on a 1.5% agarose gel. In our study, we obtained 272 polymorphic markers between IR24 and three *japonica* cultivars (Fig. 3I). The 272 newly developed markers will be useful for genetics and breeding for *indica* × *japonica* cross-derived lines together with the previously developed marker sets.

In a multiple sequence comparison, the frequency of large InDels (20‒150-bp gaps) was relatively low between the cultivated species and *O. nivara* compared to the other four species. So, we had a minor tendency to select the polymorphism between the above combinations to obtain enough polymorphic markers for all the species. In cases of difficulty in selecting polymorphism between *O. sativa* (*indica* and *japonica*) and the other species, we selected polymorphism between *indica* and the other species. For this reason, the number of polymorphic markers is overall higher between *indica* (IR24) and the other species than between *japonica* (Nipponbare) and the other species. Most of the polymorphic markers were common among the three accessions (red bars in Fig. 3 and Supplementary Fig. S2) in *O. barthii*, *O. glaberrima*, *O. longistaminata*, and *O. meridionalis*. This result suggests that three accessions within the species are genetically close to each other although they are geographically distant within the species. Furthermore, the common polymorphic markers will probably work well in other accessions of those species. In contrast, the frequency of common markers between two accessions (blue bars) and accession-specific markers (black bars) was relatively higher in *O. glumaepatula*, *O. nivara*, and *O. rufipogon*, suggesting that they are more diverse within the selected accessions in the species.

According to Kuroda et al.^55^, higher heterozygosity was observed in perennial species than in annual species or cultivated species. *O. longistaminata* and *O. rufipogon* were both perennial in nature and floral biology such as long-exserted stigma and long stamen and were contrasting from the other six AA-genome species. Several studies observed high heterozygosity in *O. longistaminata* and *O. rufipogon*, which is unlikely due to their high outcrossing rate and self-incompatibility, obtained by large reproductive organs^15,56,57^. Two accessions of *O.* *glumaepatula* (Brazil and Colombia origin) in this study showed low levels of heterozygosity. Similar results were observed by Brondani et al.^58^, in which *O. glumaepatula* populations found in Brazil showed low heterozygosity. However, one of the accessions of *O. glumaepatula* (Glum_A09) in this study exhibits a somewhat higher frequency of heterozygosity (18‒36 markers: 3.79‒7.58%). In the case of highly heterozygous wild species or accessions such as *O. longistaminata*, we need to consider both alleles to cover the whole genome of the accessions while developing ILs and CSSLs. If we transfer only one allele, we might lose superior/target alleles.

In this study, we successfully developed a medium-density InDel marker set for AA-genome *Oryza* species and validated the markers. Further, we grouped the marker set for *indica* versus the specific species and also *japonica* versus the specific species. The marker name includes the genome type of the genus *Oryza* and the chromosome number with physical location. These InDel markers can be easily and simply used by scientists and breeders with common laboratory equipment. We believe that these user-friendly marker sets will be helpful for genetic analysis and breeding by using AA-genome wild rice species and we further expect active use of stored wild germplasm for rice improvement to deal with the world population increase and climate changes.

## Supplementary Information

Supplementary Information 1.

Supplementary Information 2.

## Data Availability

All data reported in this manuscript were obtained during this study and the data are presented in the manuscript files as well as in the Supplementary information files.
