# Supplementary material for: Development of a genome-wide InDel marker set for allele discrimination between rice (Oryza sativa) and the other seven AA-genome Oryza species
Source: Sci Rep. 2021 Apr 26;11:8962. doi: 10.1038/s41598-021-88533-9 (PMC8076200; doi:10.1038/s41598-021-88533-9)
Supplement: Supplementary file 1 — Supplementary Information 1. [file 41598_2021_88533_MOESM1_ESM.pdf]

Development of a genome-wide InDel marker set for allele discrimination between rice (*Oryza sativa*) and the other seven AA-genome *Oryza* species

Sherry Lou Hechanova[1], Kamal Bhattarai[1,2], Eliza Vie Simon[1,2], Graciana Clave[1], Pathmasiri Karunarathne[1,2], Eok-Keun Ahn[3], Charng-Pei Li[4], Jeom-Sig Lee[3], Ajay Kohli[1], N. Ruaraidh Sackville Hamilton[1], Jose E. Hernandez[2], Glenn B. Gregorio[2], Kshirod K. Jena[1,6], Gynheung An[5] & Sung-Ryul Kim[1]*

[1] Gene Identification and Validation Group, Genetic Design and Validation Unit, International Rice Research Institute (IRRI), Los Baños, Laguna 4031, Philippines

[2] Institute of Crop Science (ICropS), College of Agriculture and Food Science, University of the Philippines Los Baños (UPLB), Laguna 4031, Philippines

[3] National Institute of Crop Science, Rural Development Administration (RDA), Suwon 16429, Republic of Korea

[4] Taiwan Agricultural Research Institute (TARI), Council of Agriculture, Taiwan

[5] Crop Biotech Institute and Graduate School of Biotechnology, Kyung Hee University, Yongin, Republic of Korea

[6] School of Biotechnology, KIIT Deemed University, Bhubaneswar, Odisha, India

*C**orrespondence:**

Sung-Ryul Kim

Tel: +63 2 580 5600; Email: s.r.kim@irri.org

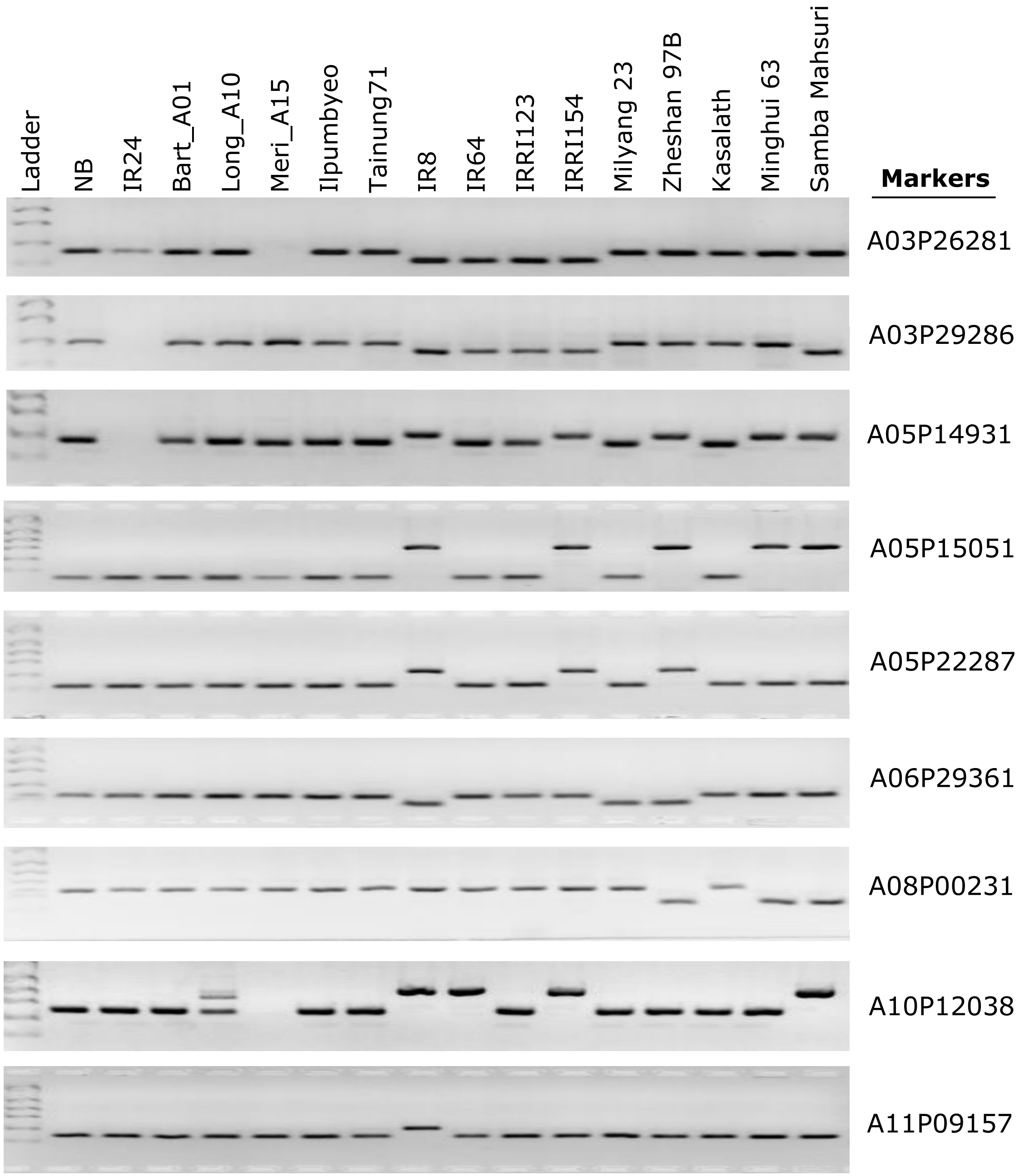

Supplementary Figure S1. Agarose gel images of the markers showing polymorphism in other cultivar backgrounds. The monomorphic markers in the 1st PCR were re-tested in other cultivar backgrounds including *japonica* varieties (Ilpumbyeo and Tainung71) and *indica* cultivars (the others).

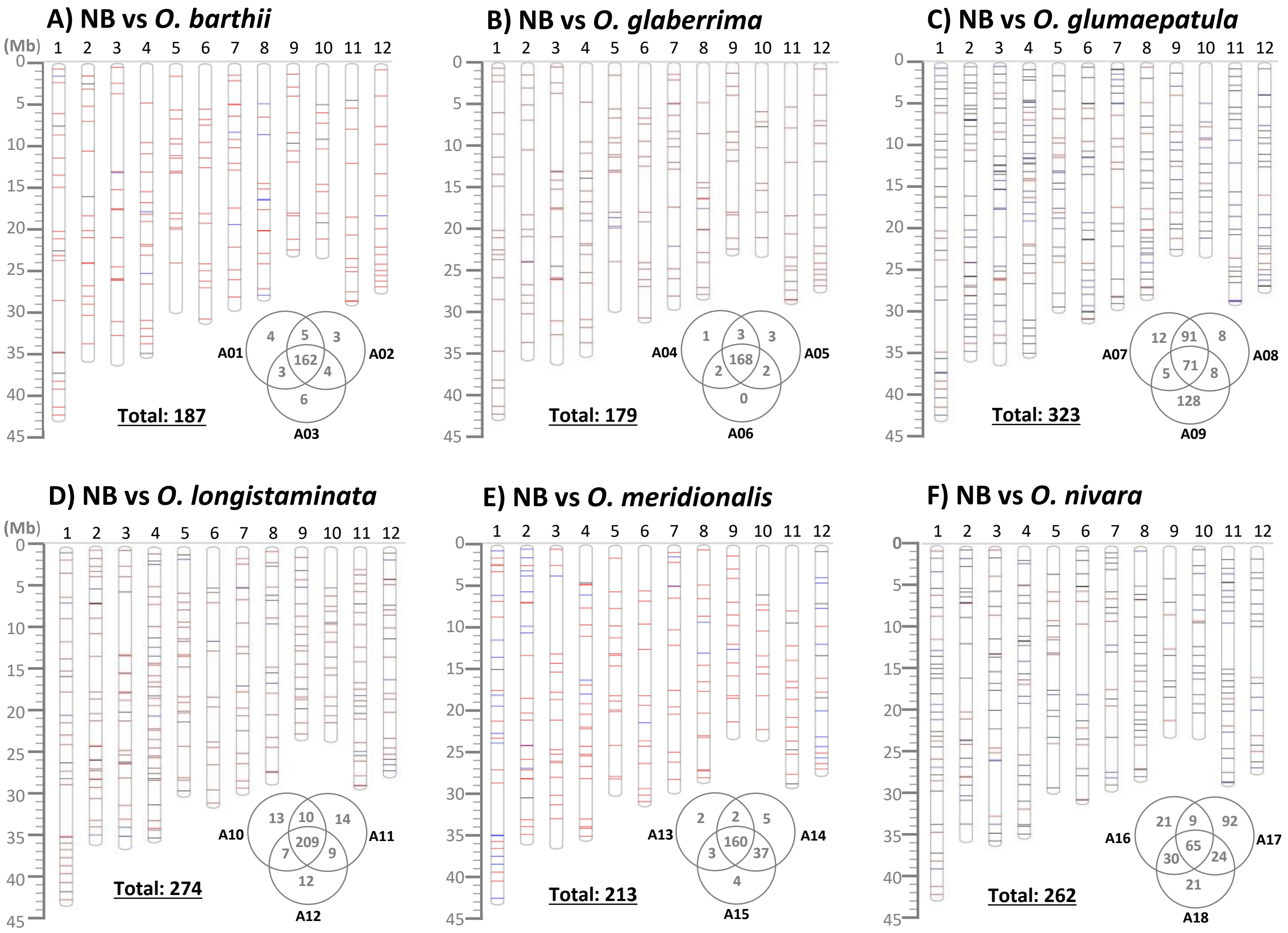

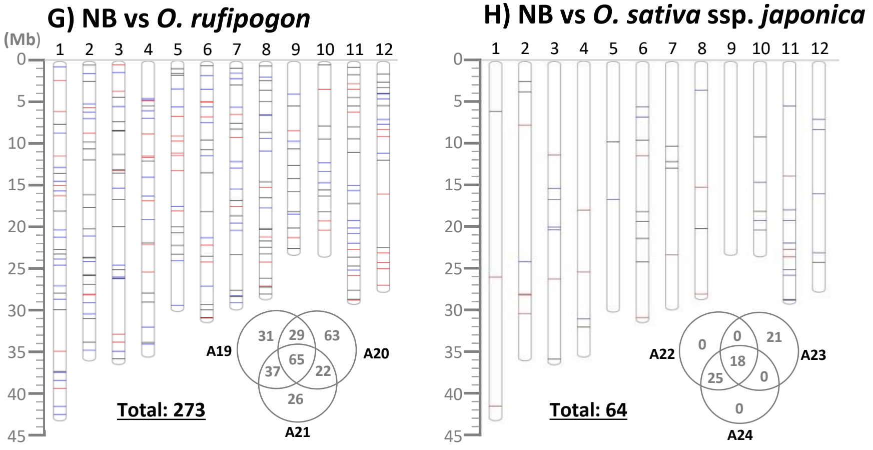

Supplementary Figure S2. Physical locations of the polymorphic markers between Nipponbare and the other seven AA-genome species. The selected polymorphic markers showing polymorphism between Nipponbare and *O. barthii* (A), *O. glaberrima* (B), *O. glumaepatula* (C), *O. longistaminata* (D), *O. meridionalis* (E), *O. nivara* (F), *O. rufipogon* (G), and *O. sativa* ssp. *japonica* (H), respectively. Within a species, the common polymorphic markers among three accessions and between two accessions are highlighted by red and blue bar respectively and the accession specific polymorphic markers are depicted with black bar.

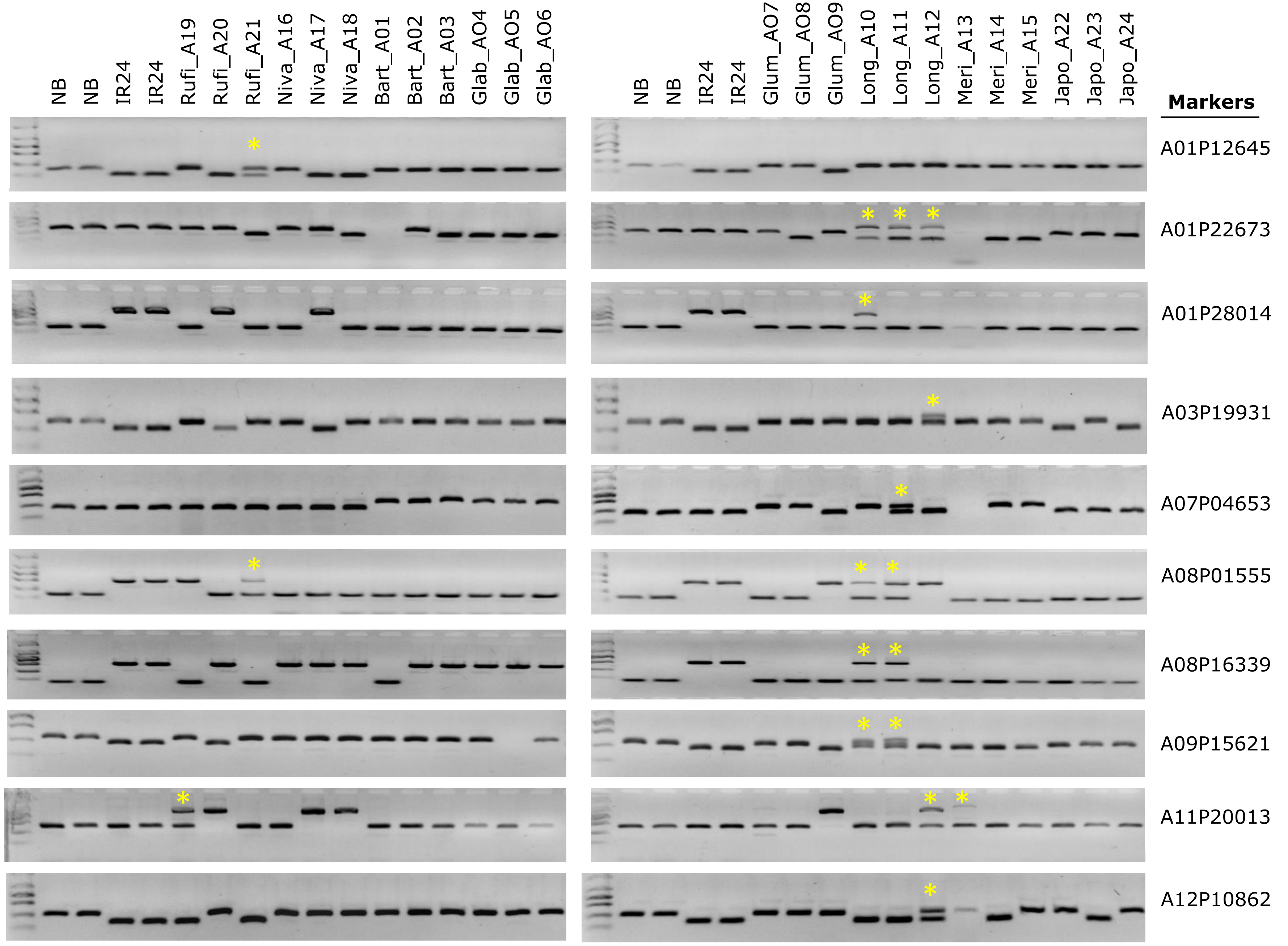

Supplementary Figure S3. Example gel images of the markers showing heterozygosity. Heterozygosity is highlighted by asterisks (*).

Original gel images

1. Original gel images of Fig. 2

2. Original gel images of Fig. S1

3. Original gel images of Fig. S3

1. Original gel images of Fig. 2

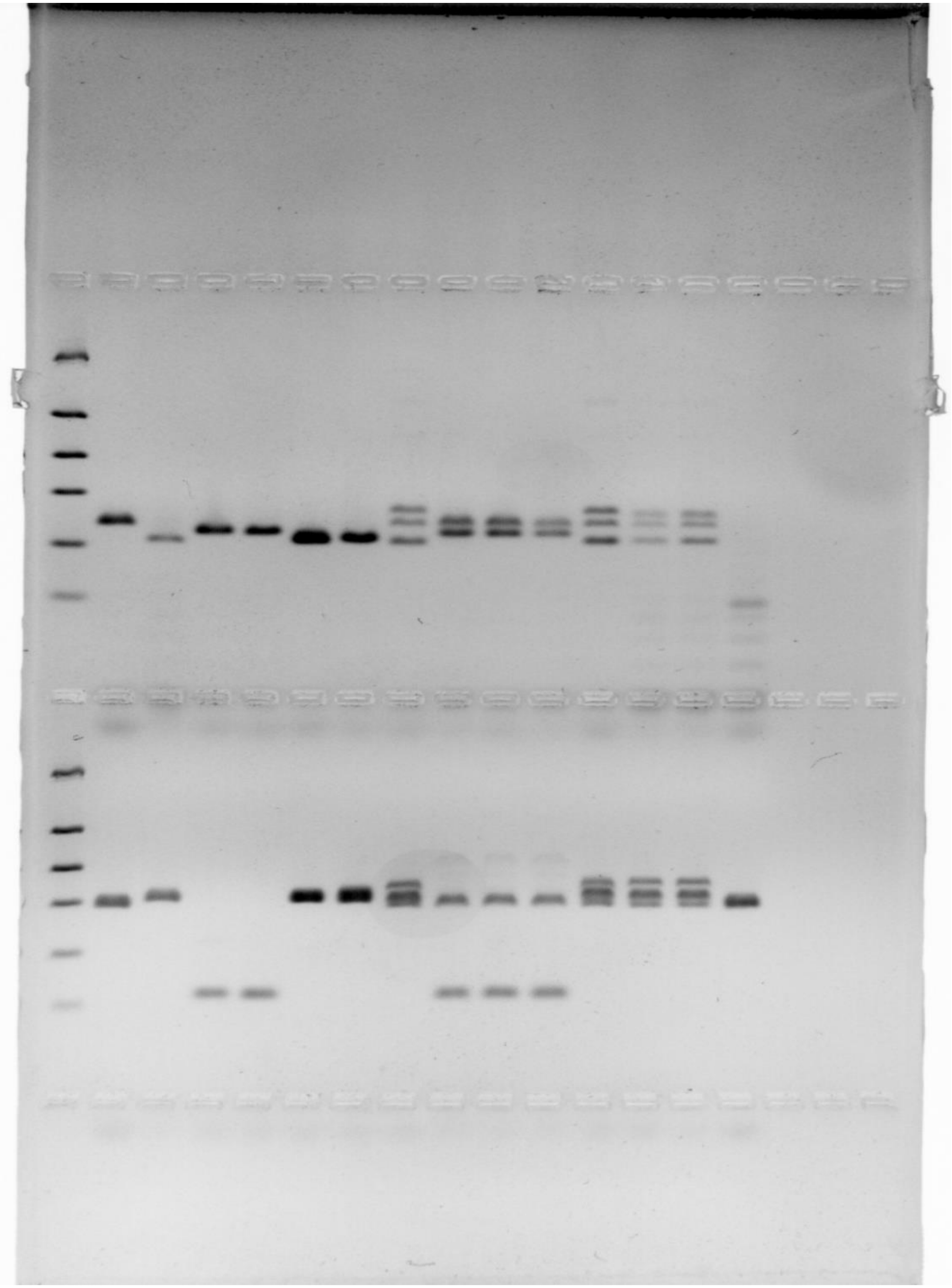

A01P11231

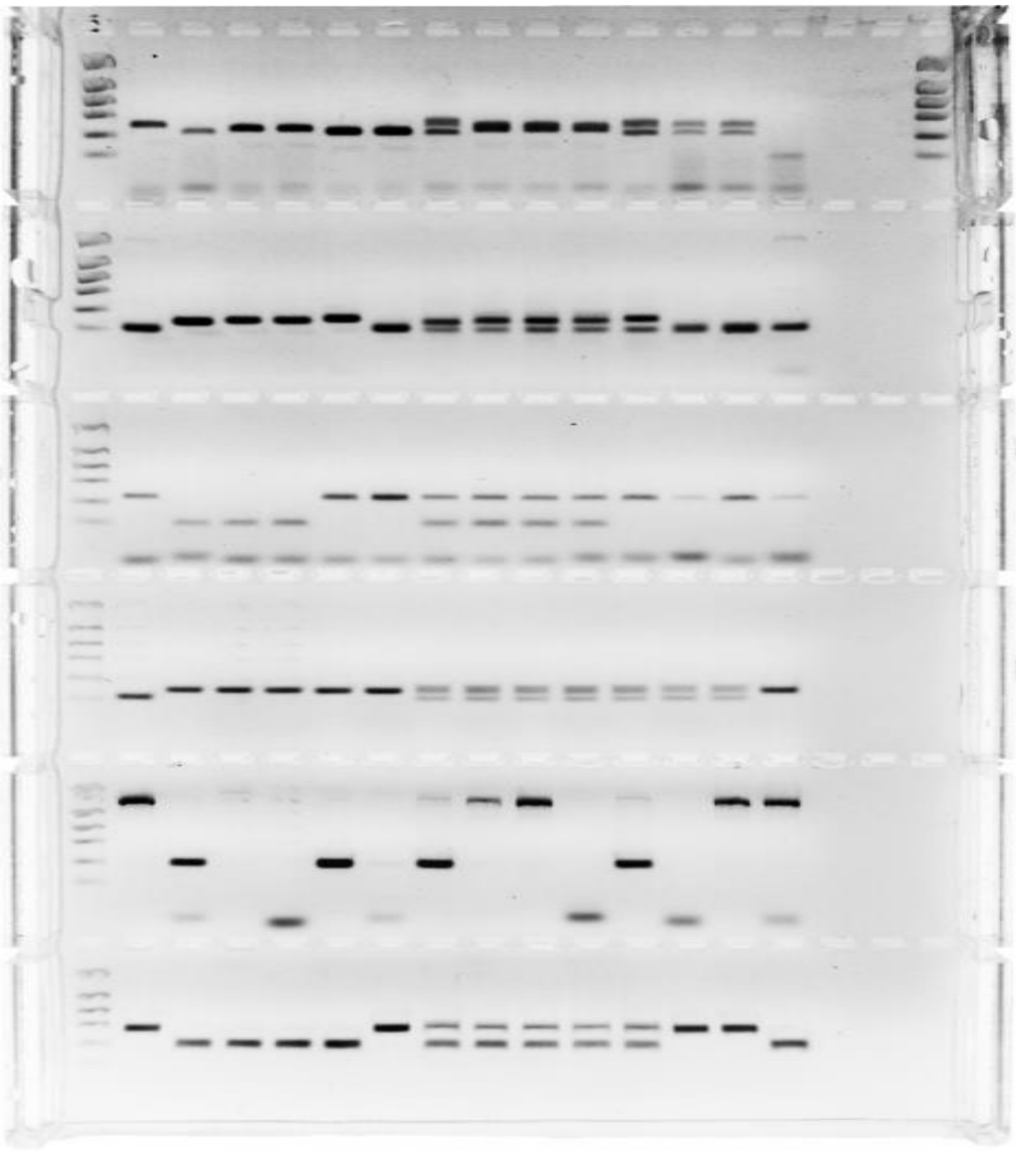
A02P20144
A03P31378
A04P29134
A06P07137

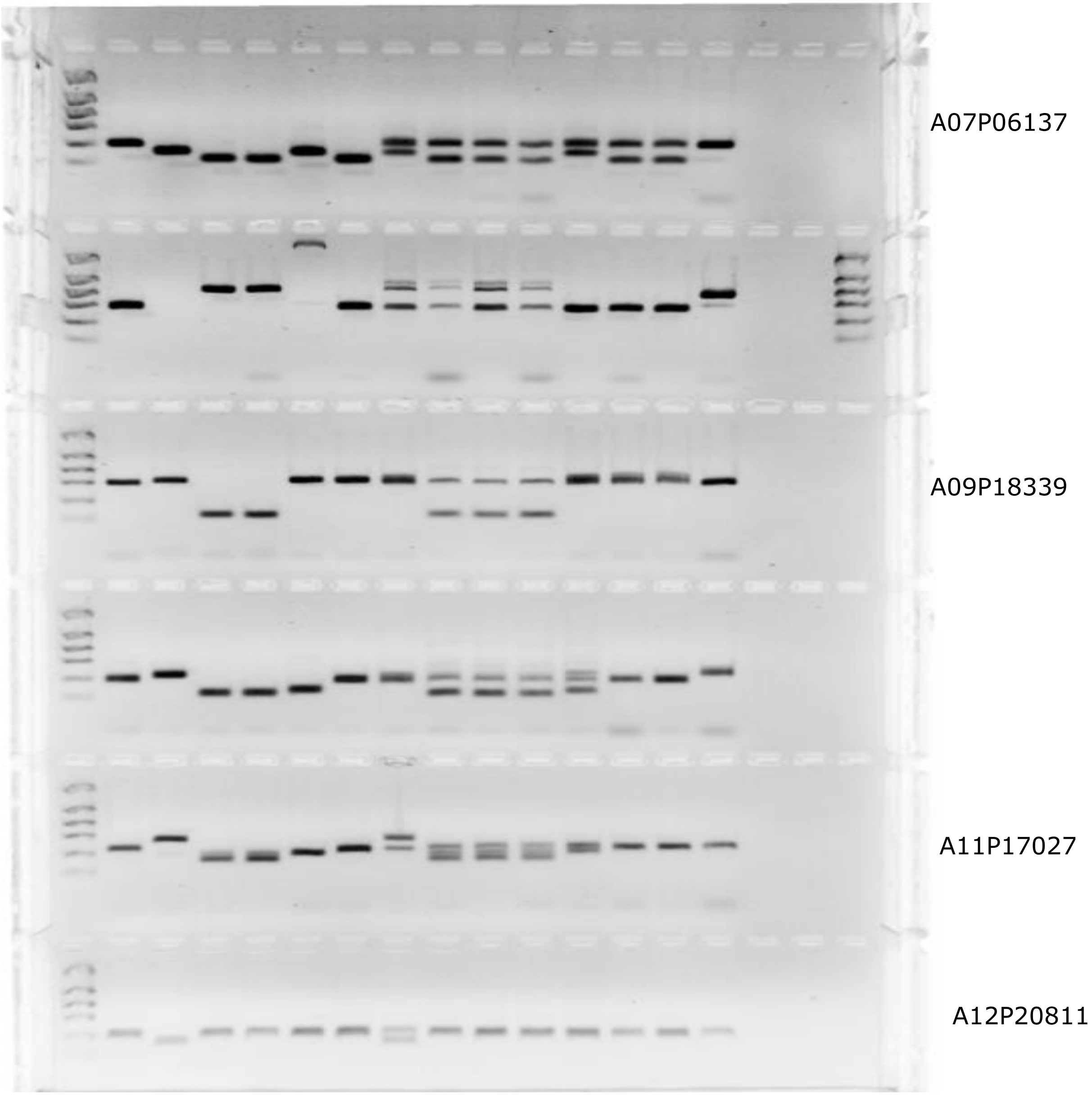
A07P06137
A09P18339
A11P17027
A12P20811

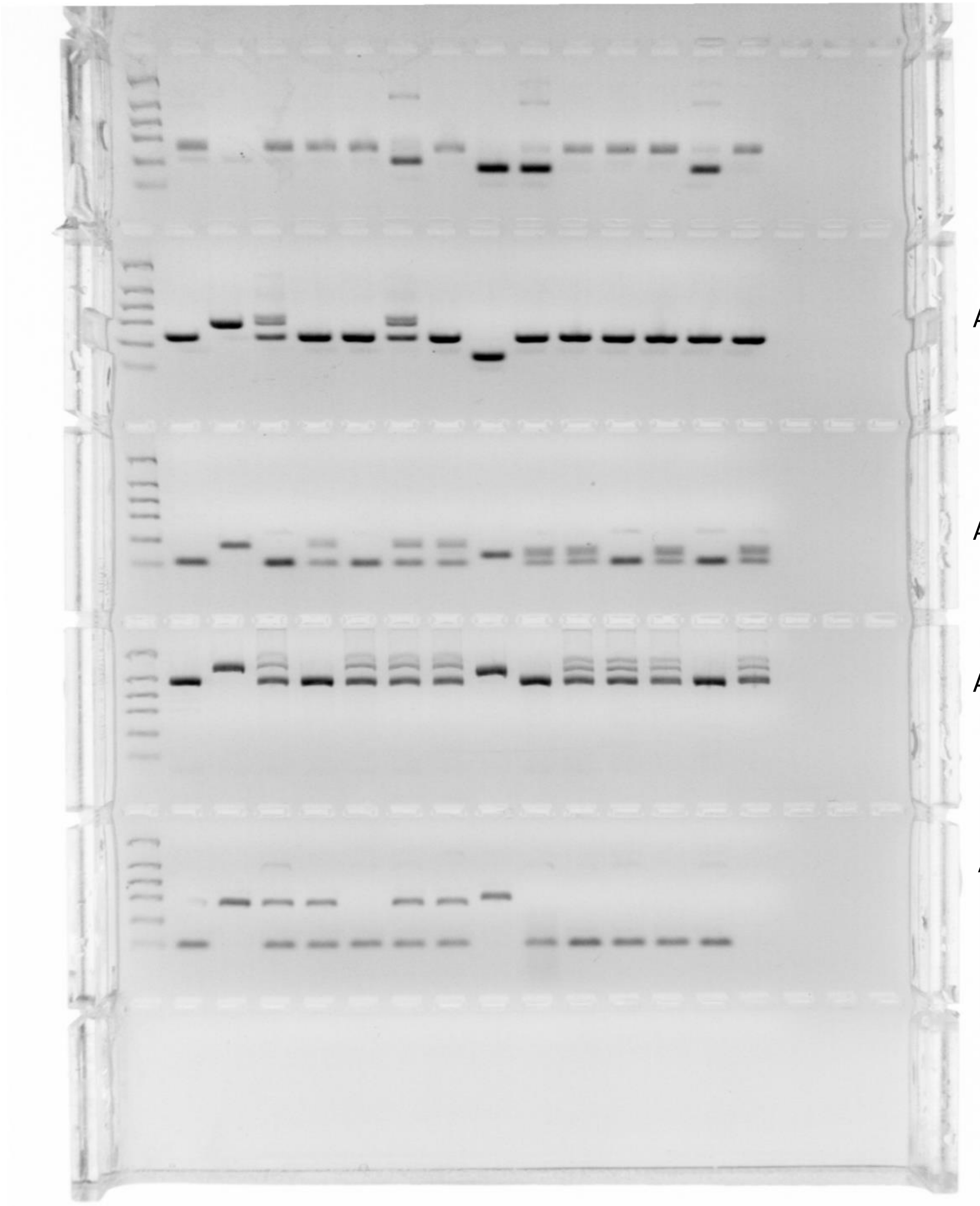

A02P28238

A03P07075

A05P18026

A08P19324

2. Original gel images of Fig. S1

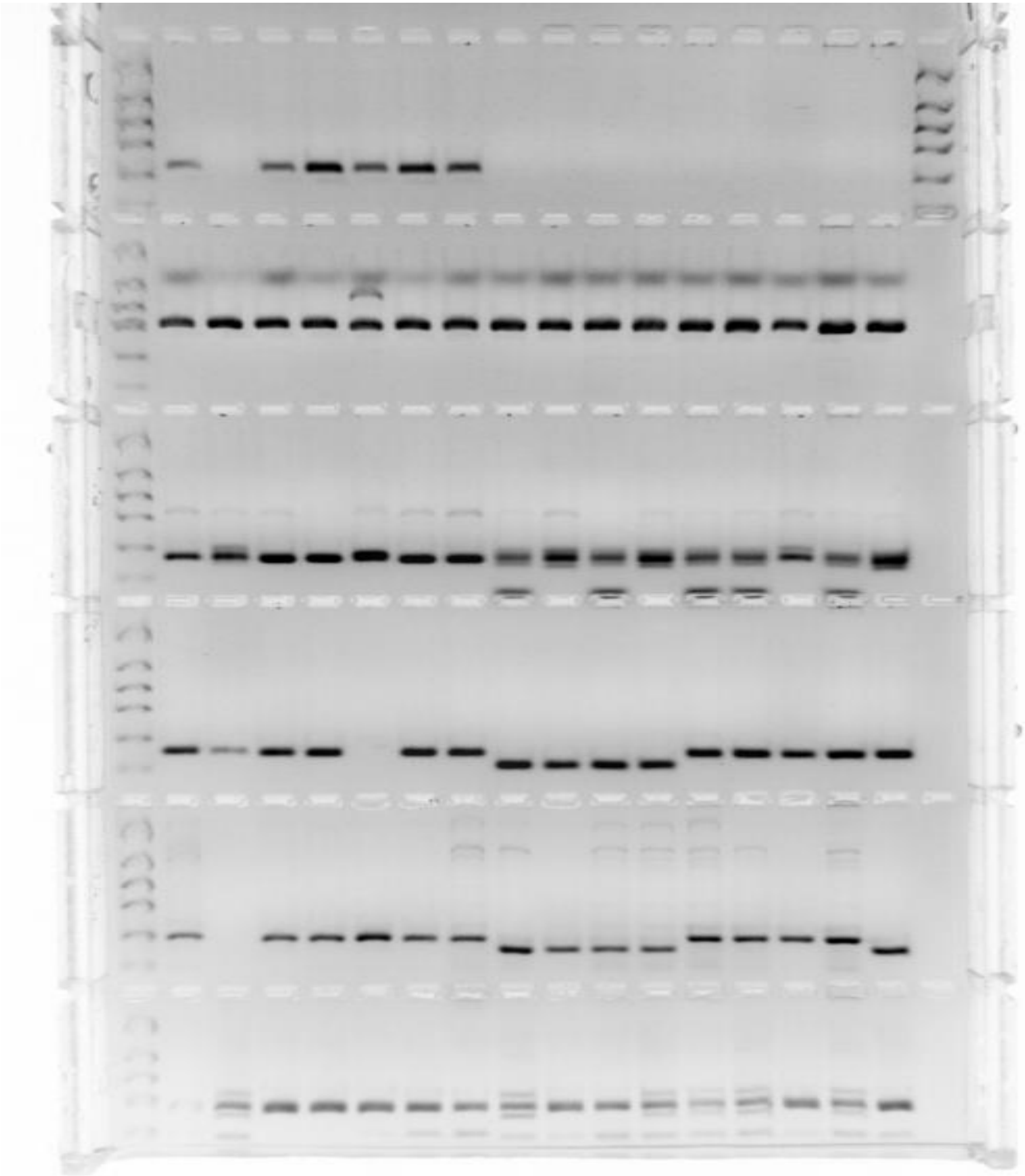

A03P26281

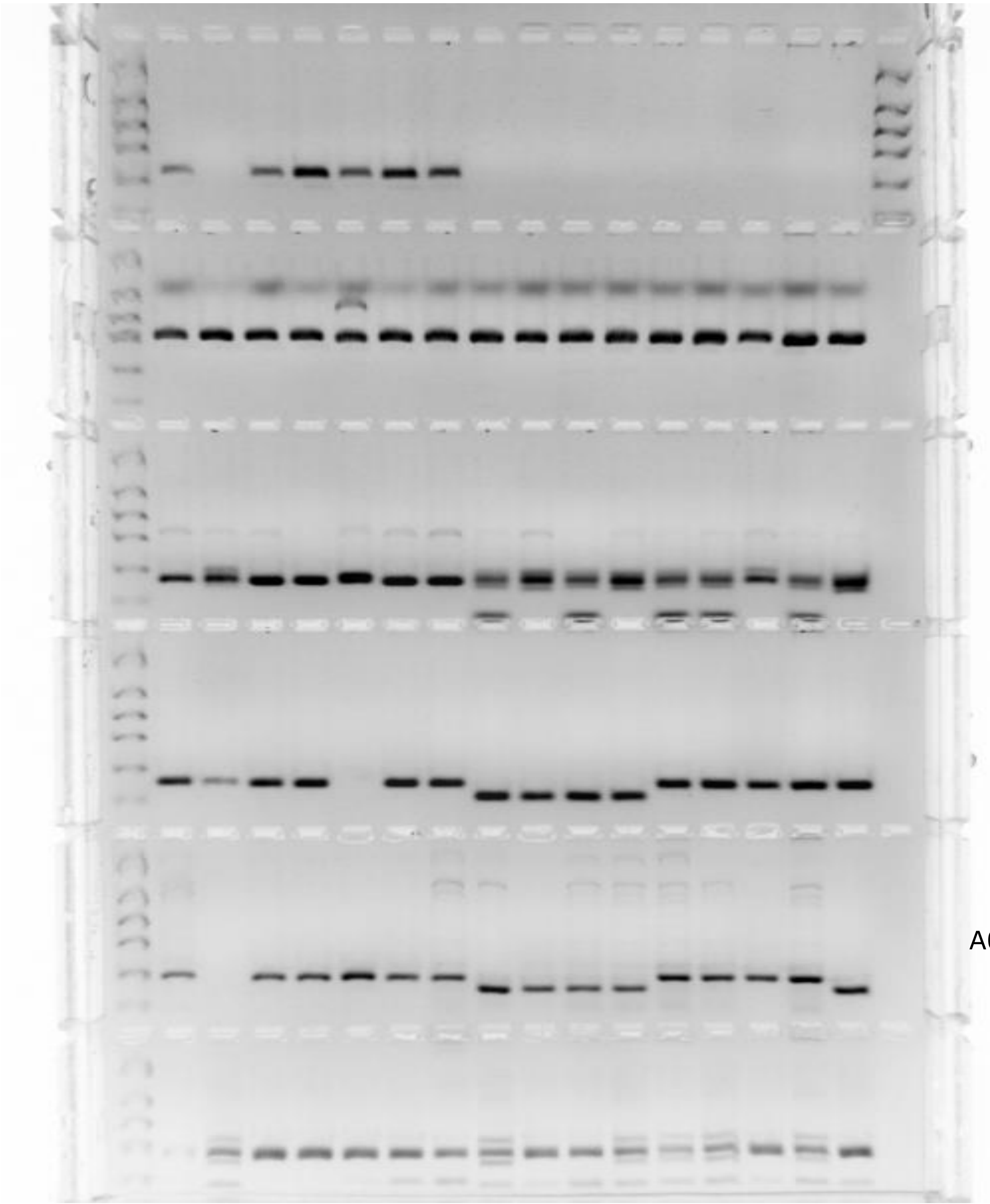

A03P29286

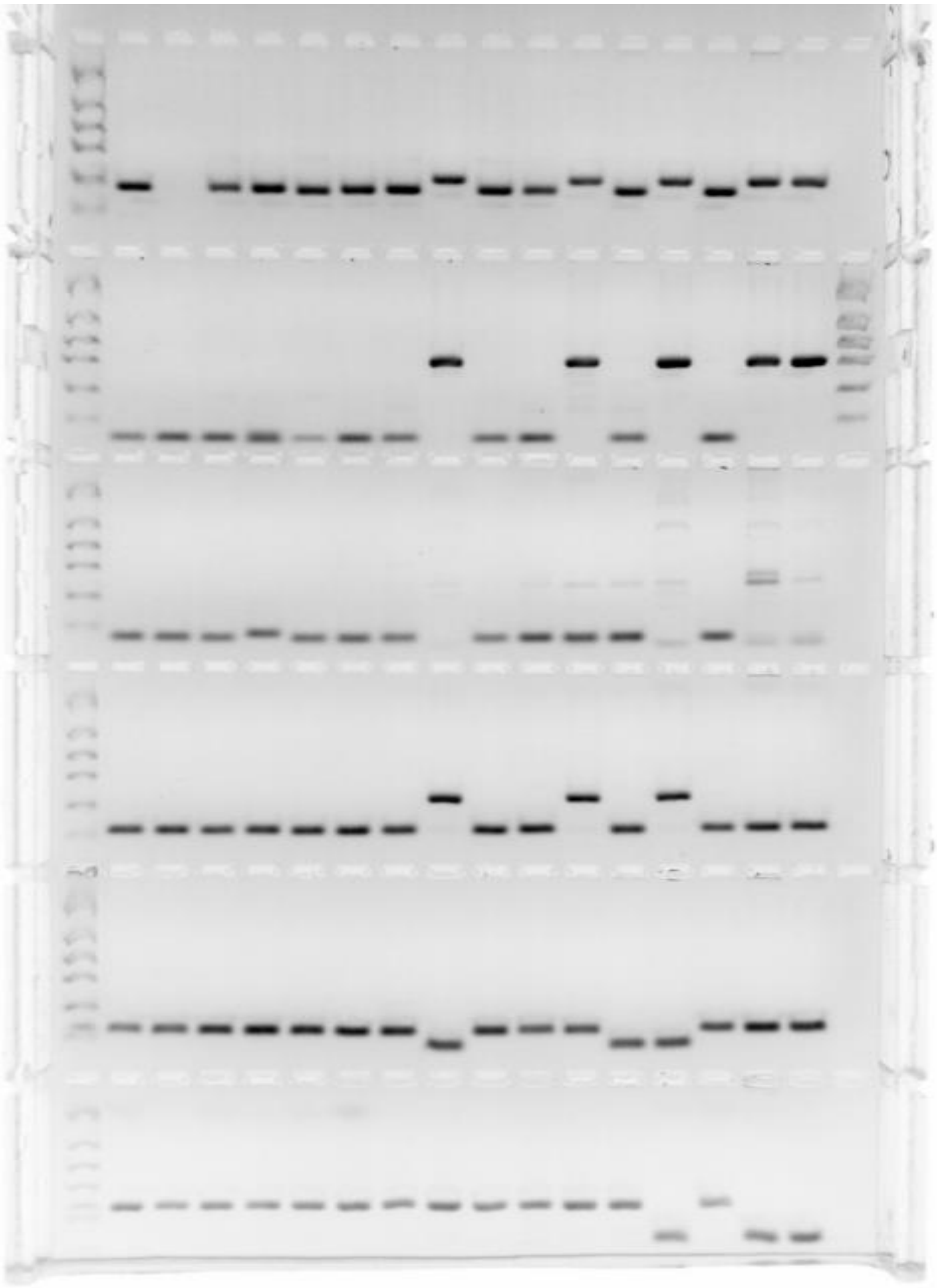

A05P14931

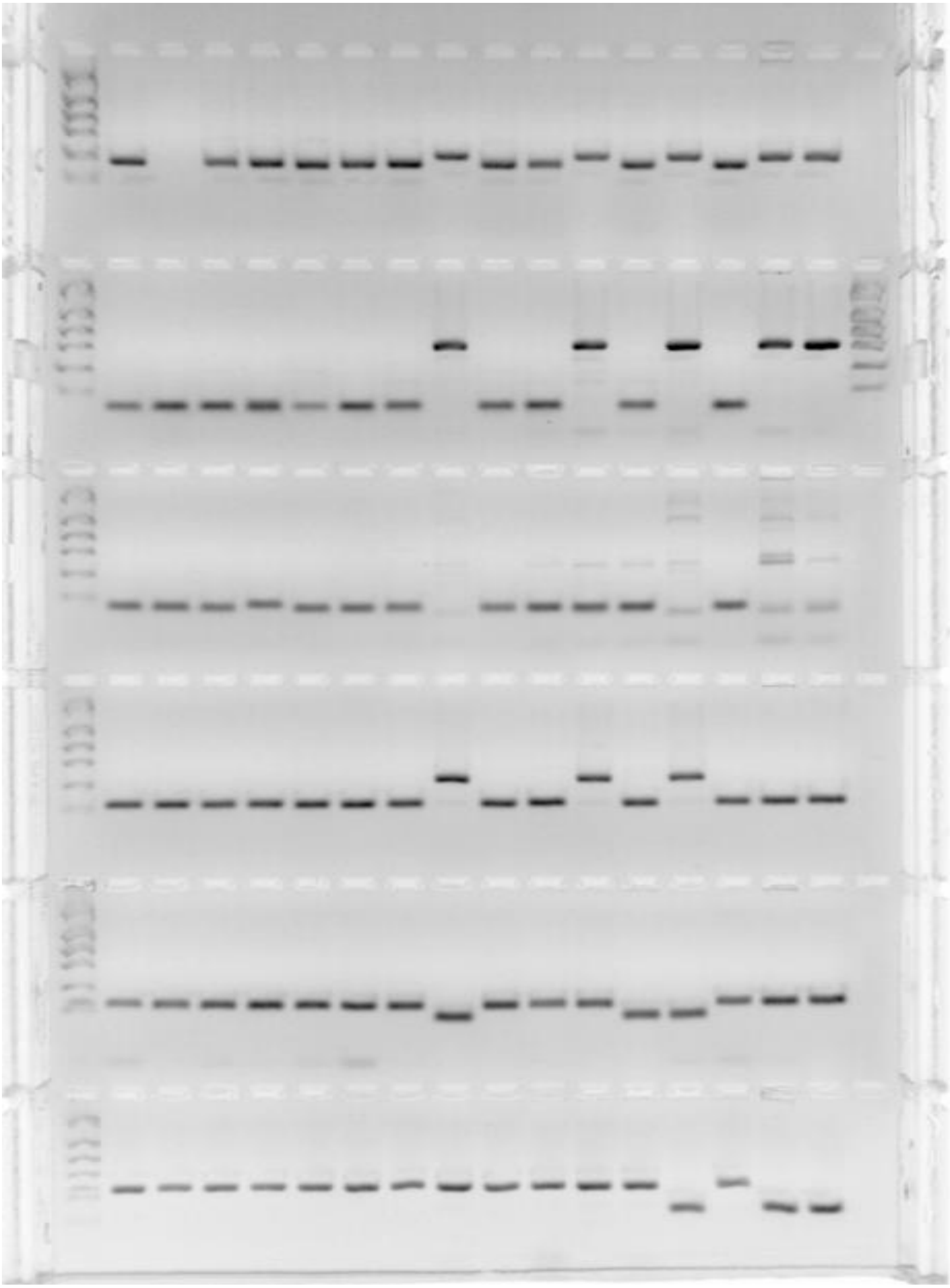

A05P15051

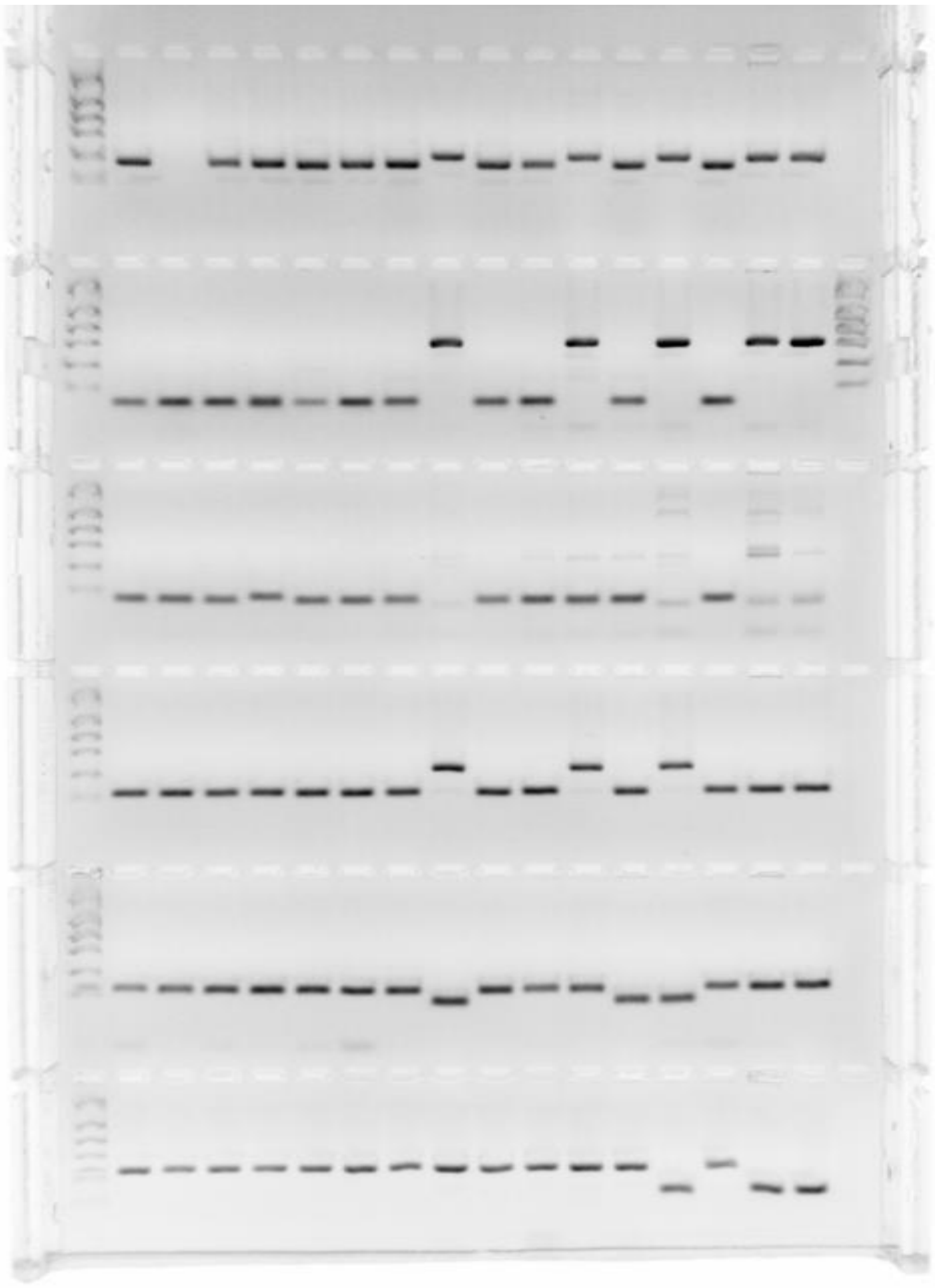

A05P22287

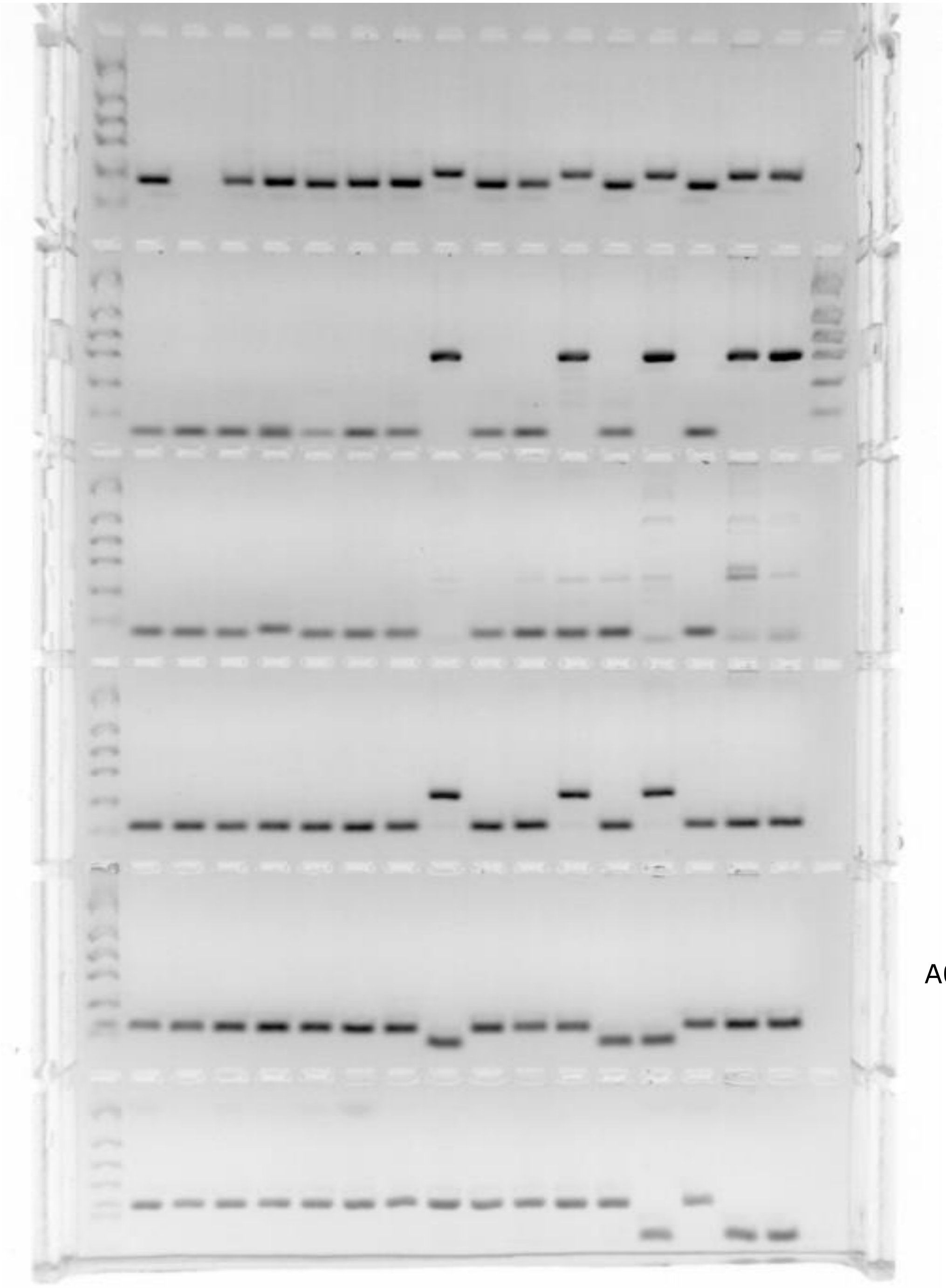

A06P29361

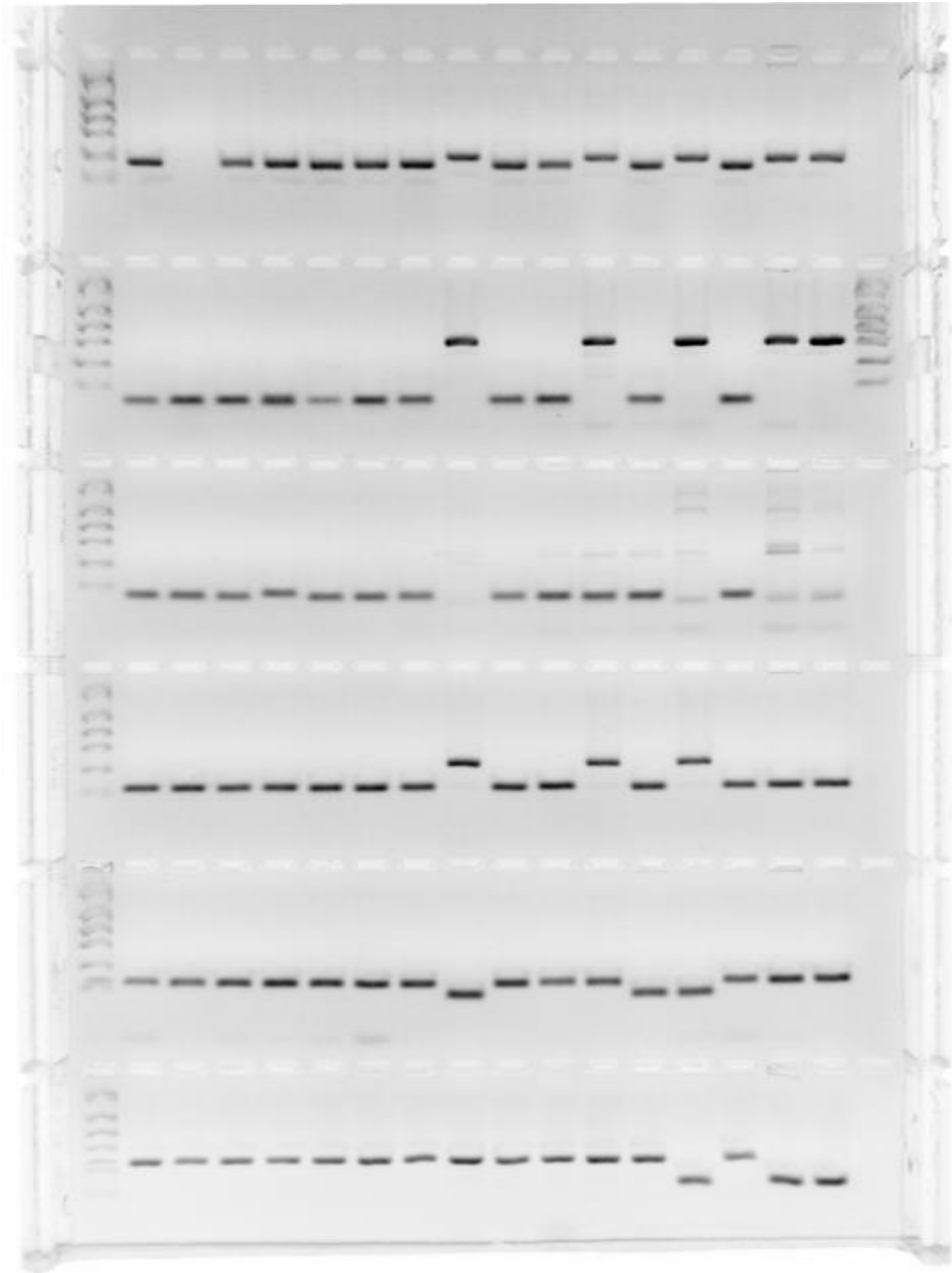

A08P00231

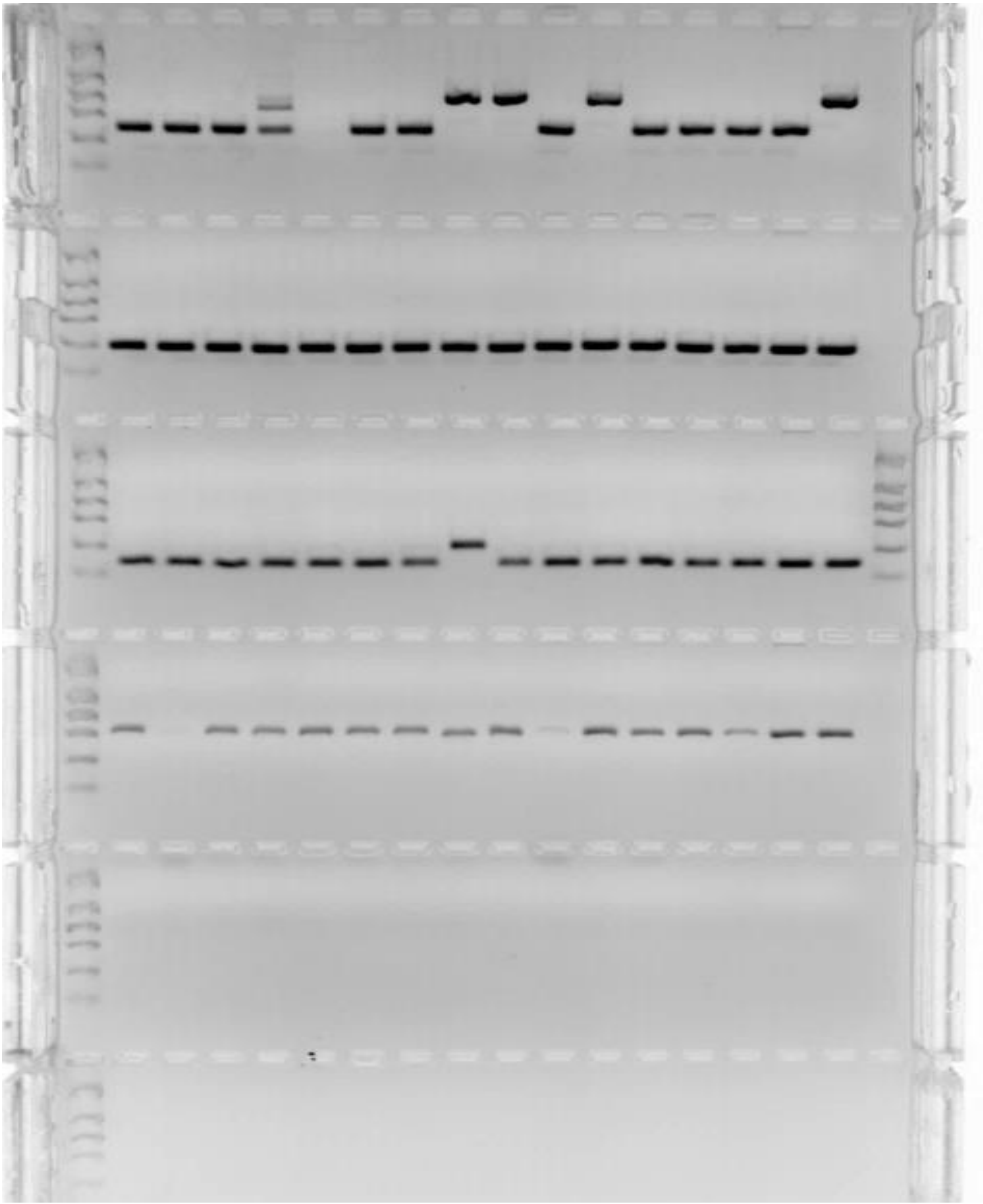

A10P12038

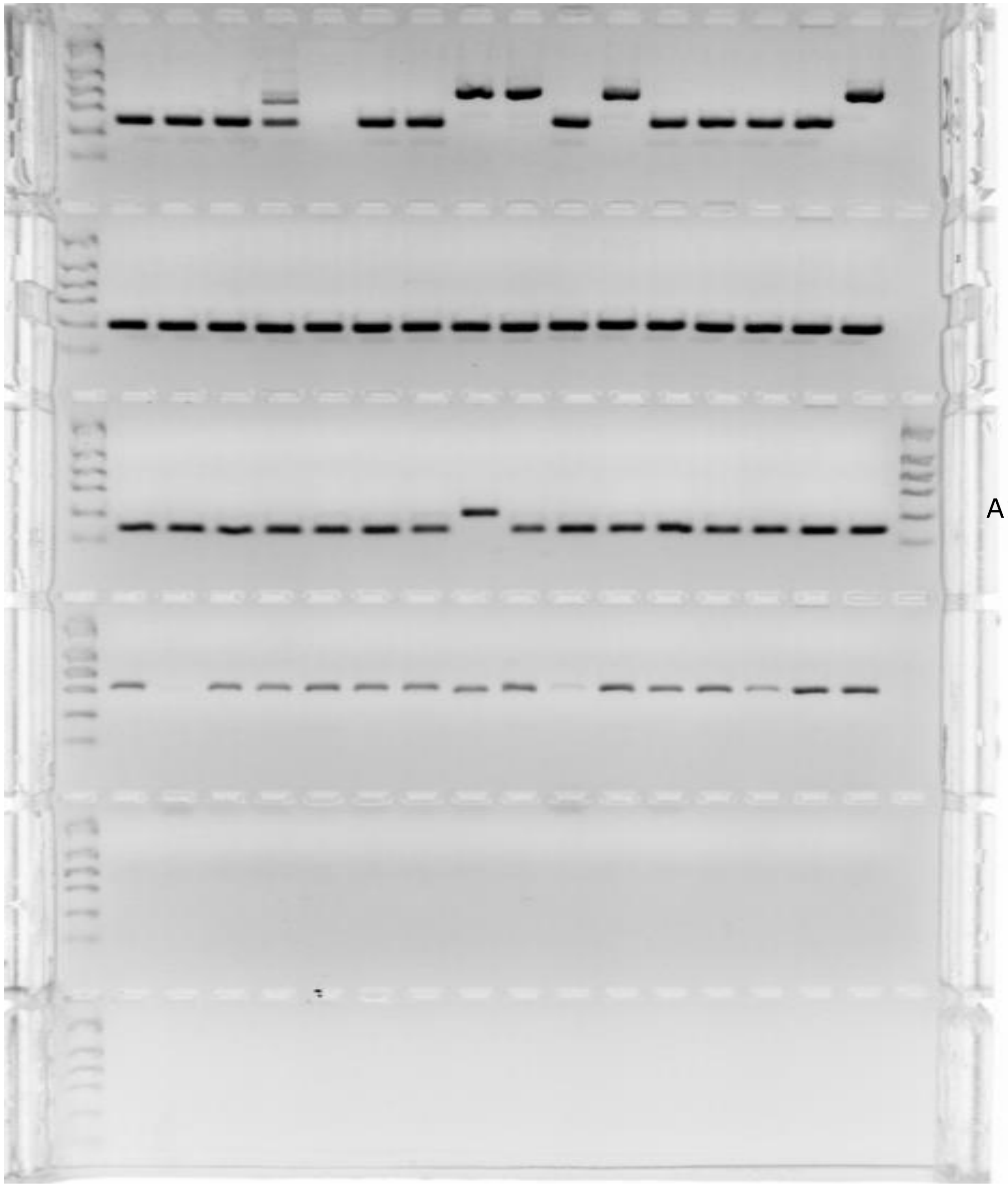

A11P09157

3. Original gel images of Fig. S3

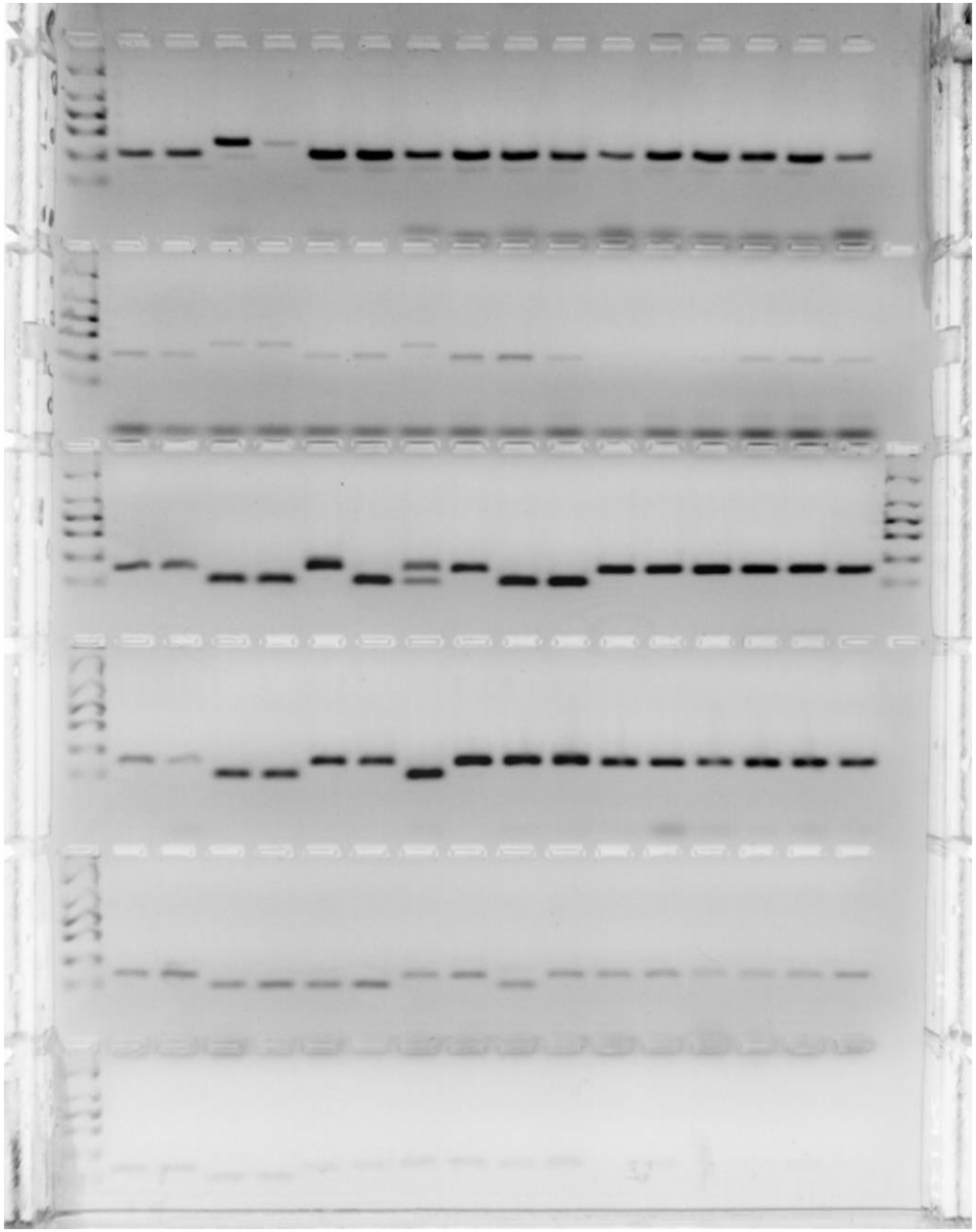

A01P12645

A01P12645

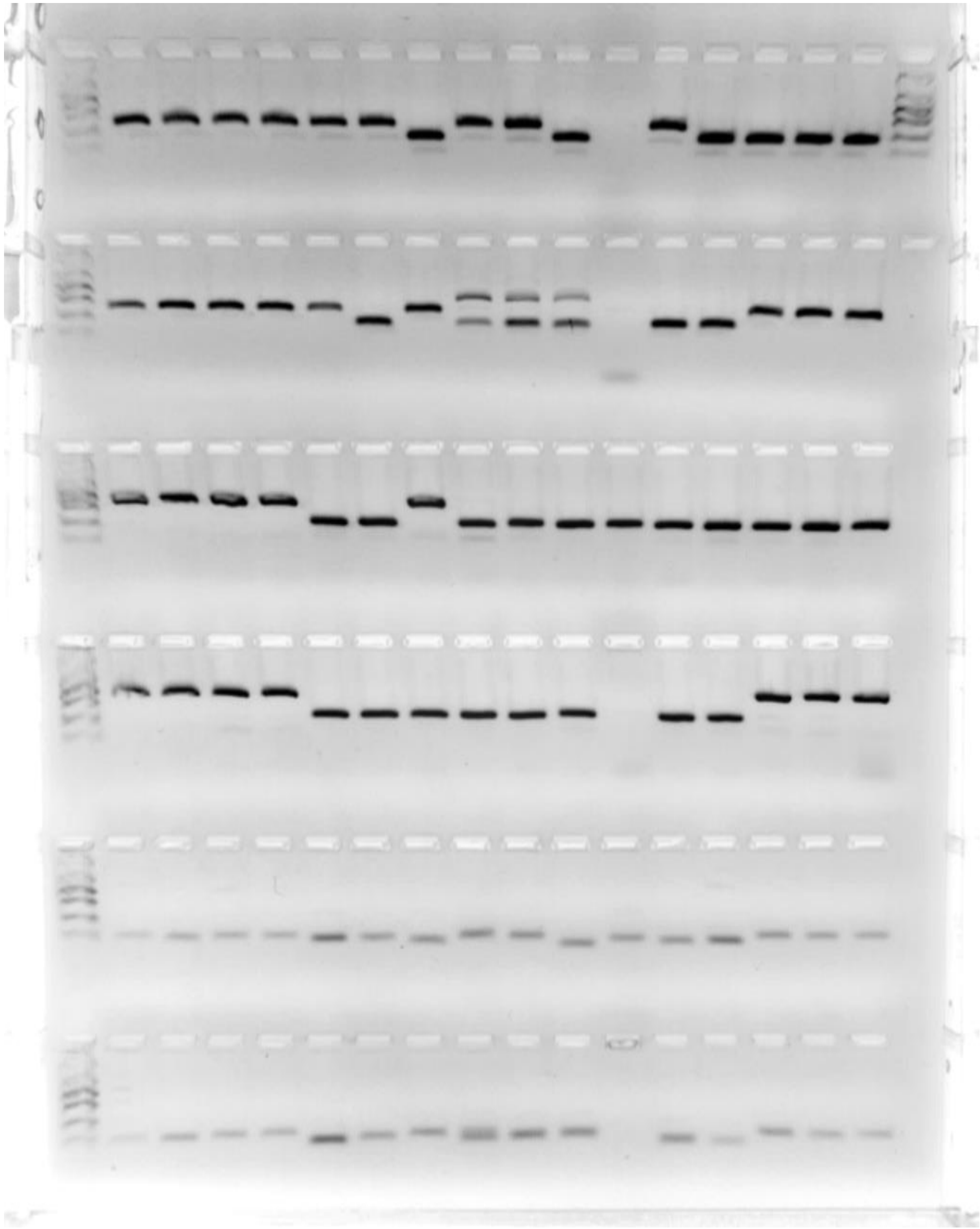

A01P22673

A01P22673

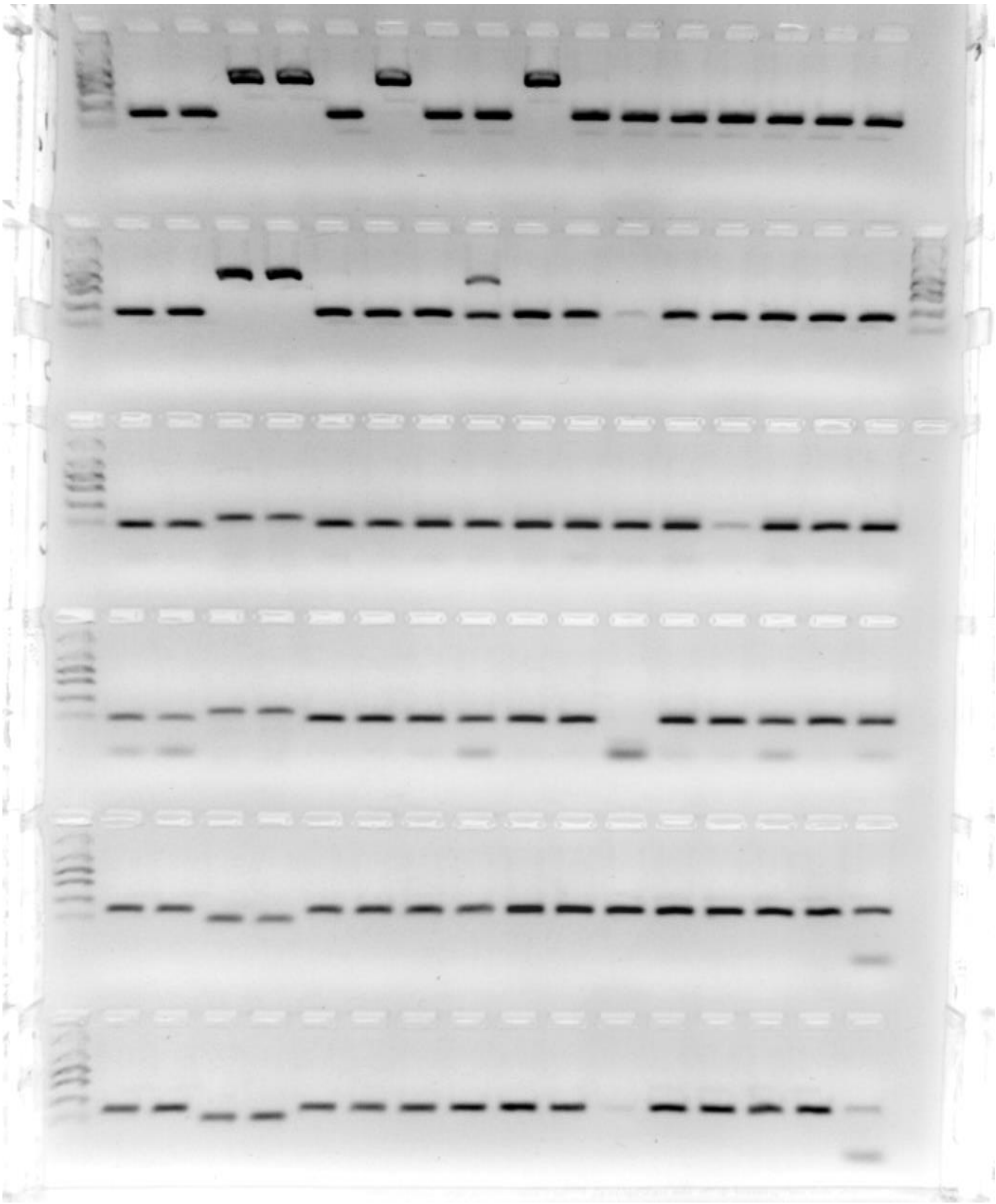
A01P28014

A01P28014

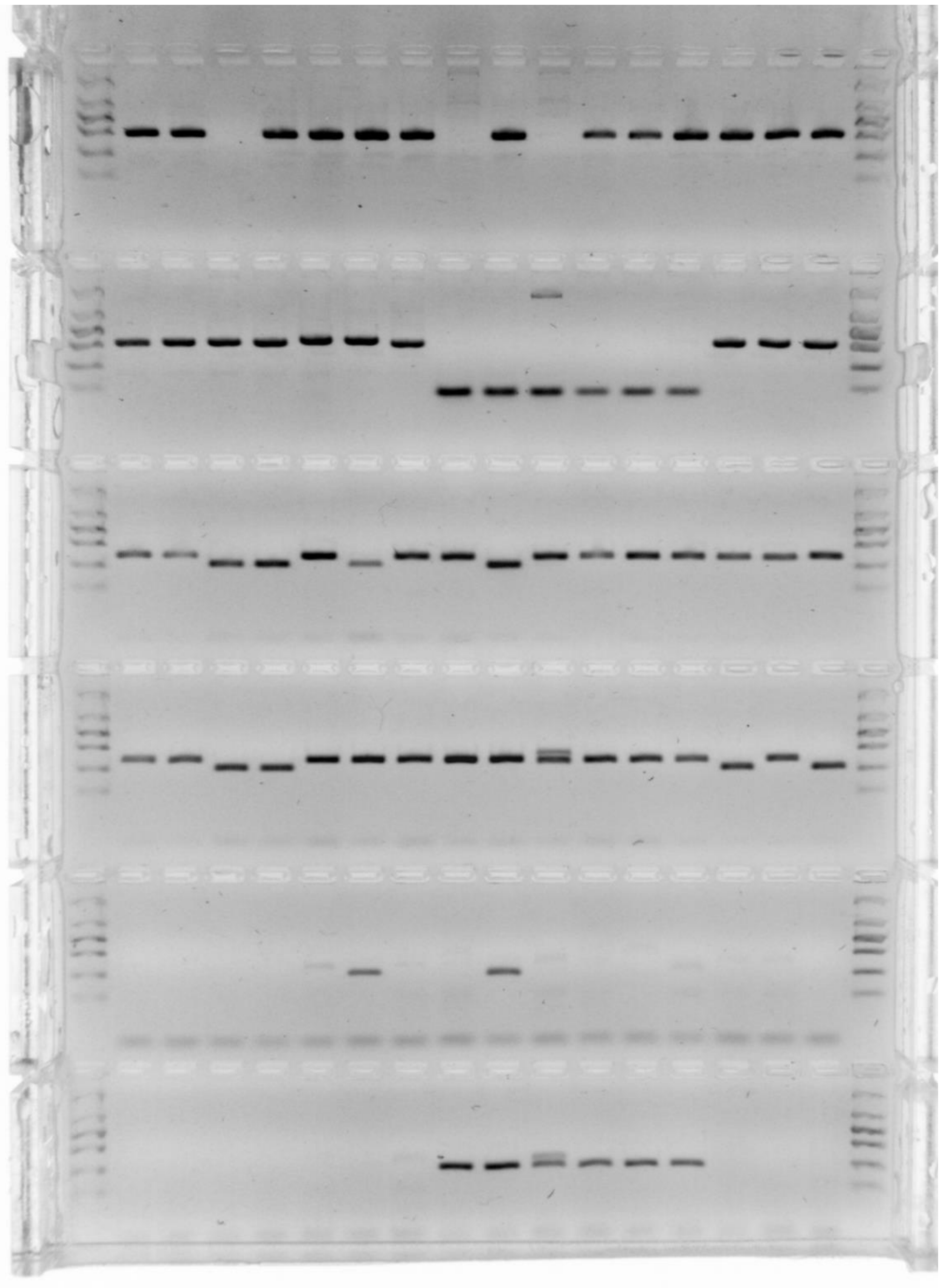

A03P19931

A03P19931

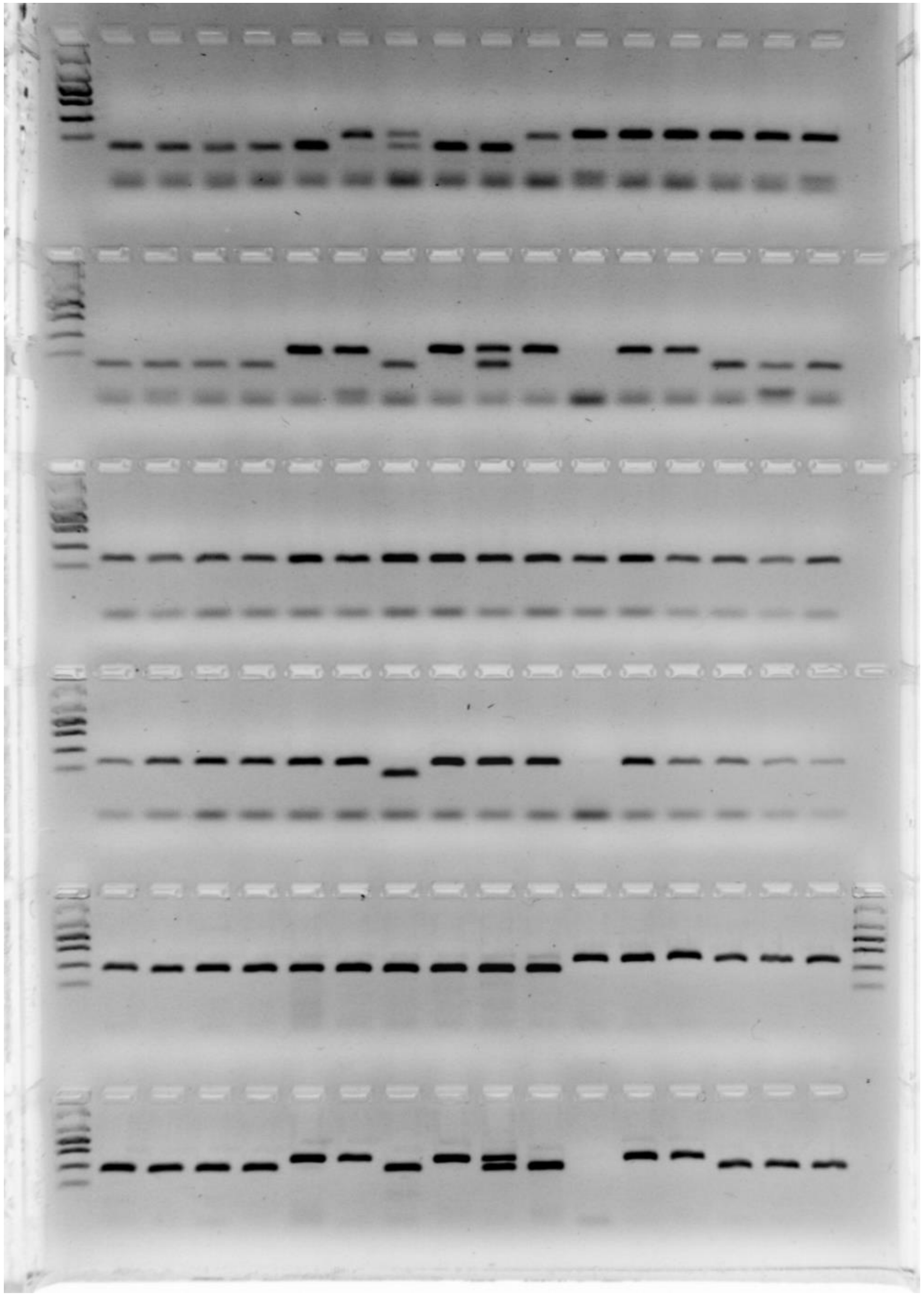

A07P04653

A07P04653

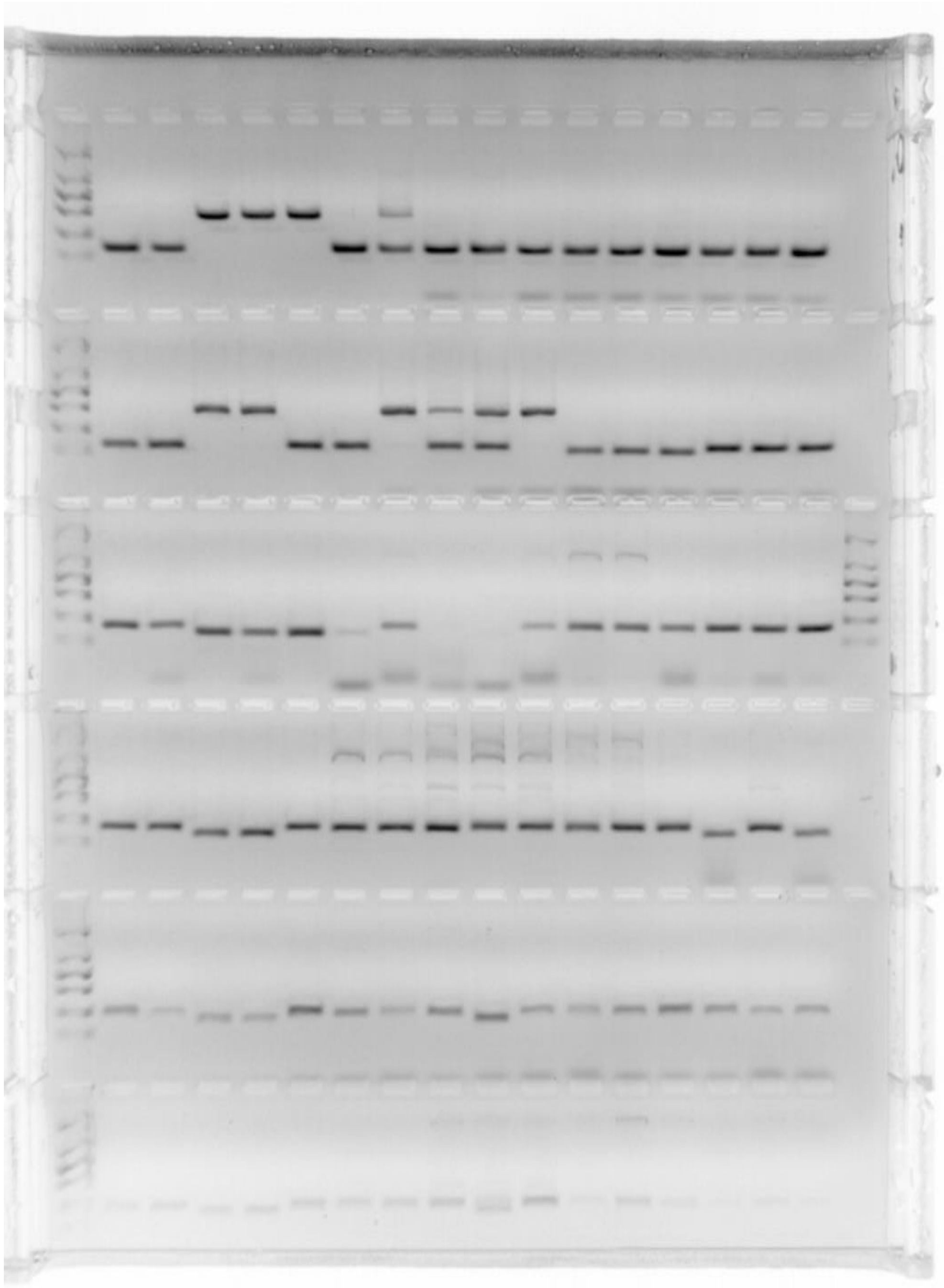

A08P01555

A08P01555

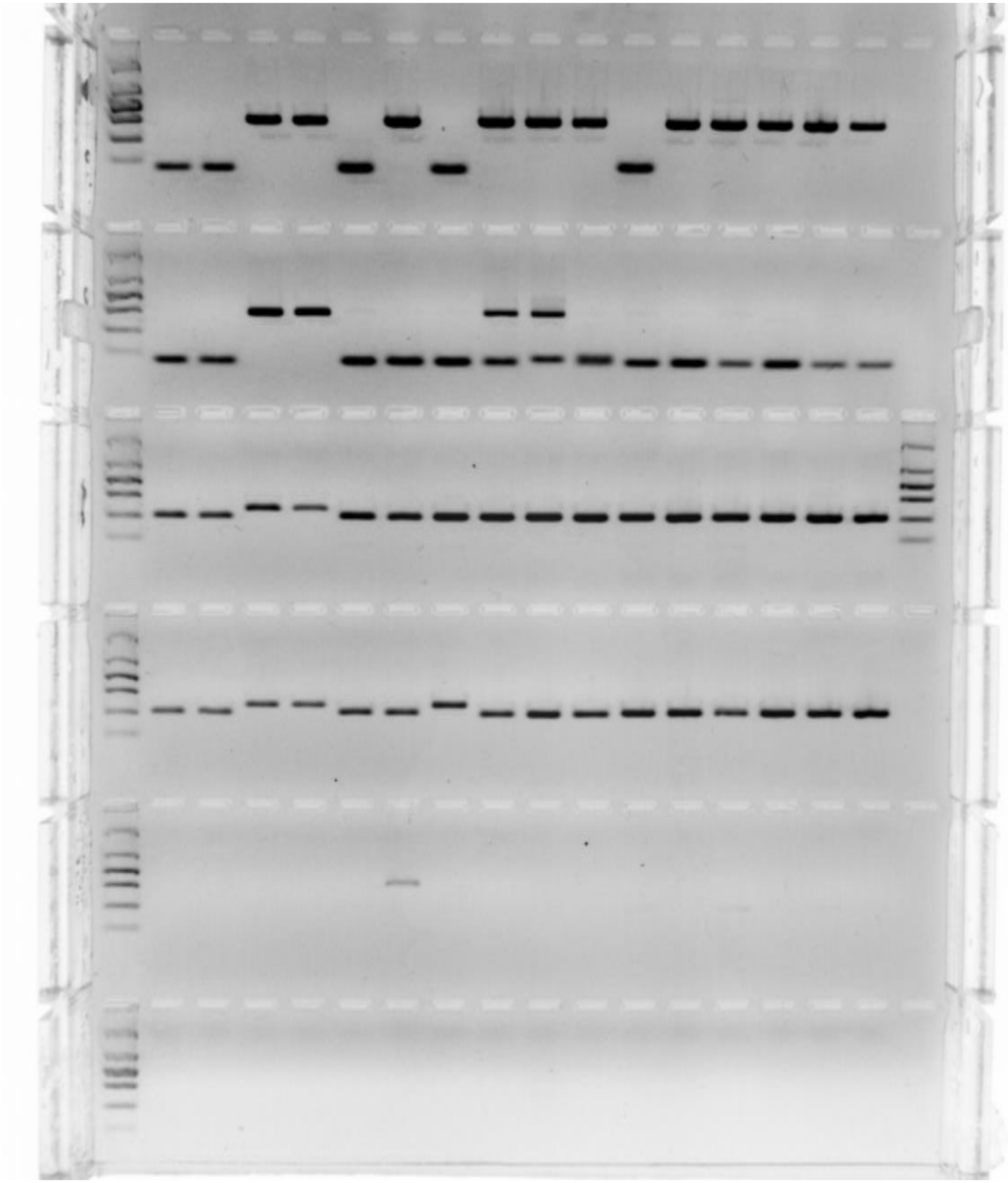

A08P16339

A08P16339

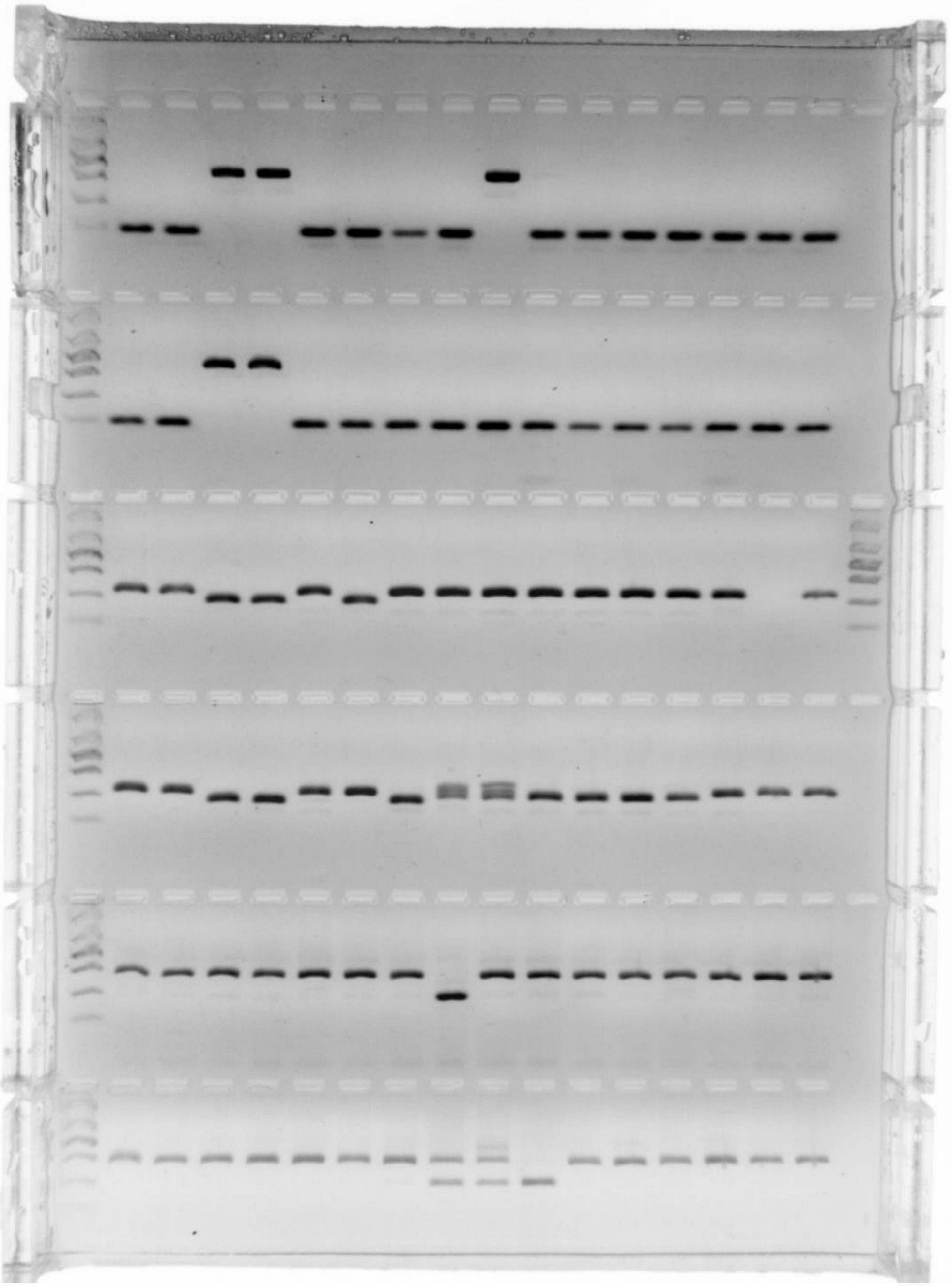

A09P15621

A09P15621

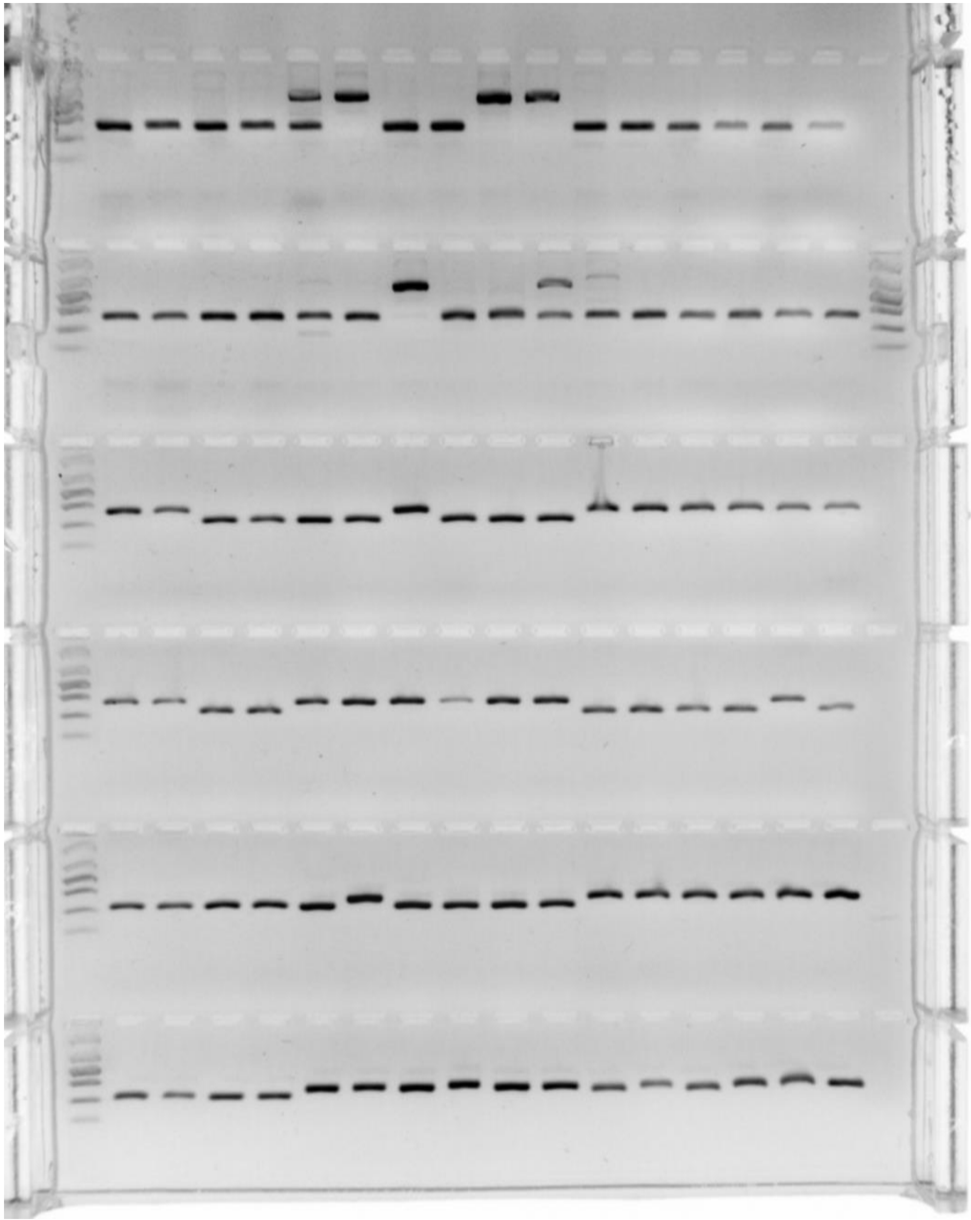

A11P20013

A11P20013

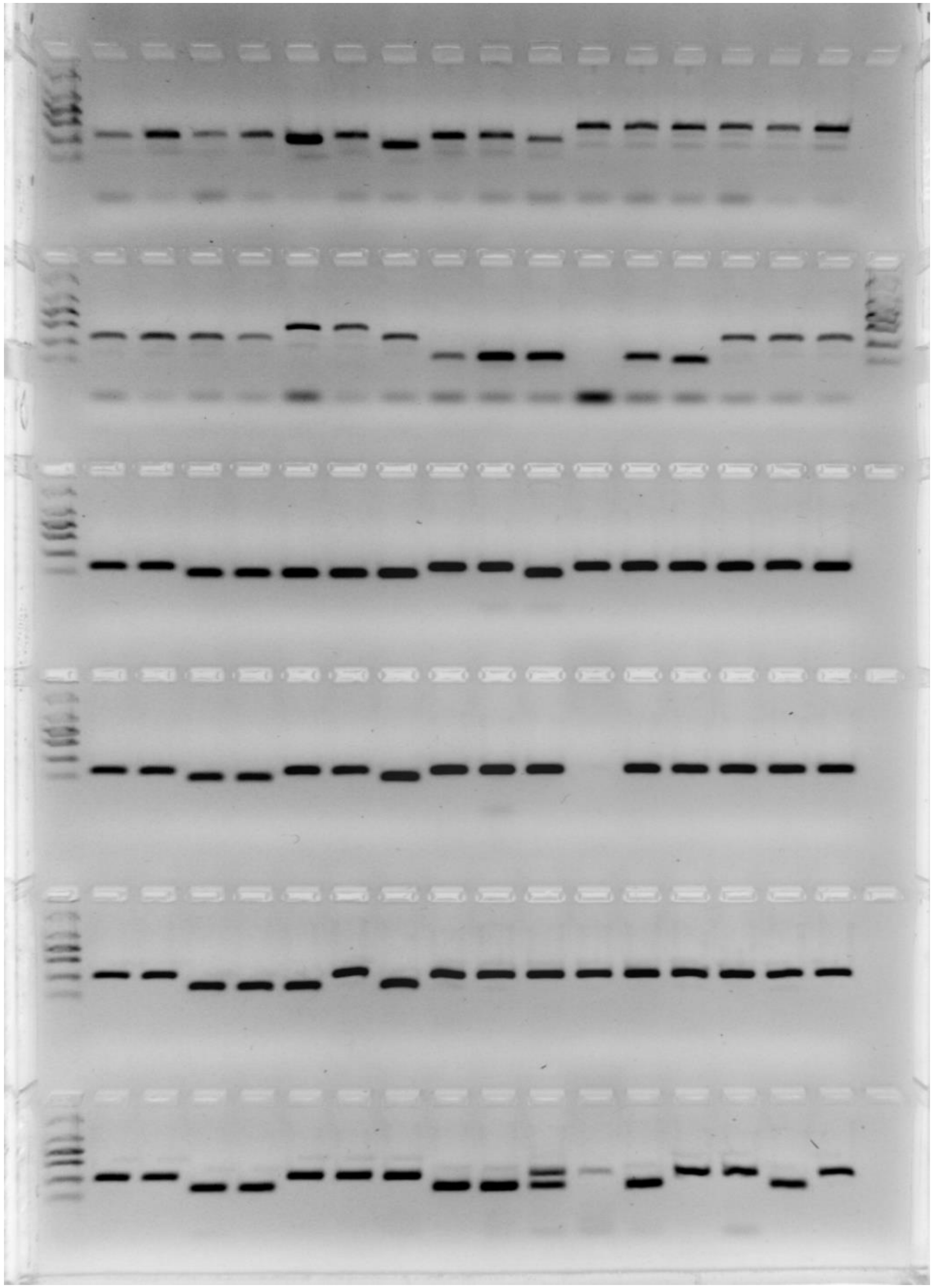

A12P10862

A12P10862
